# STAT3 phosphorylation at serine 727 activates specific genetic programs and promotes clear cell renal cell carcinoma (ccRCC) aggressiveness

**DOI:** 10.1038/s41598-023-46628-5

**Published:** 2023-11-09

**Authors:** J. Arévalo, I. Campoy, M. Durán, S. Nemours, A. Areny, M. Vall-Palomar, C. Martínez, G. Cantero-Recasens, A. Meseguer

**Affiliations:** 1 Renal Physiopathology Group, Vall d’Hebron Research Institute, Passeig Vall d’Hebron 119-129, 08035 Barcelona, Spain; 2 Molecular Oncology Group, Biodonostia Health Research Institute, Paseo Dr. Begiristain, s/n, 20014 San Sebastián, Spain; 3 Vascular and Renal Translational Research Group, Lleida Institute for Biomedical Research Dr. Pifarré Foundation (IRBLleida), Av. Alcalde Rovira Roure, 80, 25198 Lleida, Spain; 4 Unitat de Bioquímica de Medicina, Departament de Bioquímica i Biologia Molecular, Universitat Autònoma de Barcelona, Bellaterra, Spain

**Keywords:** Cancer models, Molecular medicine

## Abstract

The signal transducer and activator of transcription 3 (STAT3) is a transcription factor mainly activated by phosphorylation in either tyrosine 705 (Y705) or serine 727 (S727) residues that regulates essential processes such as cell differentiation, apoptosis inhibition, or cell survival. Aberrant activation of STAT3 has been related to development of nearly 50% of human cancers including clear cell renal cell carcinoma (ccRCC). In fact, phosho-S727 (pS727) levels correlate with overall survival of ccRCC patients. With the aim to elucidate the contribution of STAT3 phosphorylation in ccRCC development and progression, we have generated human-derived ccRCC cell lines carrying STAT3 Y705 and S727 phosphomutants. Our data show that the phosphomimetic substitution Ser727Asp facilitates a pro-tumoral phenotype in vitro*,* in a Y705-phosphorylation-independent manner. Moreover, we describe that STAT3 phosphorylation state determines the expression of different subsets of target genes associated with distinct biological processes, being pS727-dependent genes the most related to cellular hallmarks of cancer. In summary, the present study constitutes the first analysis on the role of overall STAT3 phosphorylation state in ccRCC and demonstrates that pS727 promotes the expression of a specific subset of target genes that might be clinically relevant as novel biomarkers and potential therapeutic targets for ccRCC.

## Introduction

Renal cell carcinoma (RCC), the third most prevalent urological cancer, accounts for approximately 3% of all new cancer cases and its incidence has been rising steadily over the last three decades^1^. Clear cell RCC (ccRCC) is the most aggressive and prevalent subtype accounting for approximately 80% of all renal cancers. Commonly asymptomatic, most RCCs are discovered incidentally on medical imaging. A great percentage of them may be treated by surgery, but 1/3 of patients will present either with locally advanced tumor or metastases, resulting in a 95% mortality rate. Moreover, one-third of organ-confined cancers treated by nephrectomy develop metastasis during the follow-up^2^. Development of macroscopic metastases is the major cause of tumor-associated deaths and prediction of metastatic disease for organ-confined tumors is of the utmost importance. Increased angiogenesis and tumor-induced immune evasion are two mechanisms by which ccRCC proliferate and metastasize. The development of targeted agents such as tyrosine-kinase inhibitors (TKI) targeting angiogenesis has changed the treatment of choice for patients with metastatic RCC^3^. New therapies have improved the median survival period of patients with advanced ccRCC, which is about 26 months^4^. More recently, promising immunotherapies’ results to block immune checkpoints such as cytotoxic T-lymphocyte–associated antigen 4 (CTLA4) and programmed cell death protein 1 (PD-1) illustrate the importance of targeting molecules that inhibit T cell-mediated antitumor immunity^5^.

A central role for metabolic reprogramming upon activation of oncogenic pathways is emerging in the modulation of cancer plasticity. Accordingly, aberrant metabolism is recurrent in ccRCC where glycolysis increases regardless of oxygen availability (Warburg effect)^6^. Another metabolic shift in ccRCC is the accumulation of cholesterol, cholesterol esters, and other lipids enclosed in intracellular lipid droplets that support energy production during rapid tumor cell growth and transformation^7,8^. Integrated multi-omics studies have revealed distinctive metabolic reprogramming signatures displaying altered levels of biochemical enzymes, substrates, metabolic intermediates, and final products that could be useful diagnostic biomarkers in ccRCC^6,9,10^.

Signal transducer and activator of transcription 3 (STAT3) is a crucial signaling mediator promoting immunosuppression in tumor-associated immune cells, as well as proliferation, survival, and angiogenesis in tumor cells^11^. Increasing evidence suggests that STAT3 plays a direct role in modulating the biology of cancer cells, through its involvement in energy metabolism, glucose, and lipid metabolism^12^. It regulates the expression of multiple genes involved in essential biological processes^13^ and is abnormally active in 50% of all liquid and solid tumors^12,14^. STAT3 canonical activation pathway relies on the phosphorylation of its tyrosine 705 (Y705) in response to cytokines, growth factors, and -under pathological conditions-oncogenes^15^. Once Y705 is phosphorylated, formation of STAT3 dimers is facilitated leading to nuclear translocation, DNA binding, and initiation of gene transcription^16^. Although phospho-Y705 (pY705) is the main post-translational modification (PTM) described in STAT3, it can also be phosphorylated at its serine 727 (S727), which has emerged as a relevant PTM regulating STAT3 activity in a non-canonical manner. Recent reports have shown that STAT3 can exert its pro-tumoral effect via phosho-S727 (pS727) in different types de tumors, in a pY705-independent manner^17–23^, suggesting that pS727 is required to promote the expression of a subset of genes different from those regulated by pY705^24,25^. Moreover, pS727 allows the incorporation of STAT3 into the inner mitochondrial membrane to support oxidative phosphorylation (OXPHOS) maximizing the utility of glucose sources and resulting in increased production of ATP that provides additional energy for rapid proliferation of tumor cells^26^. Contrary to mitochondrial pS727, pY705 favors glycolysis over OXPHOS^27^.

Previous work from our group showed statistically significant differences in overall survival rates between patients with high versus low pS727 levels, even when exhibiting the same clinic and pathologic tumor features; indicating that pS727 is an independent factor clinically relevant^28^. Similar results were found in a second independent cohort, which reinforces the role of pS727 in ccRCC progression and severity^29^. Association of pS727 levels with overall survival of ccRCC patients from two independent cohorts with 5–10 years of follow-up upon surgery^28,29^, prompts us towards the identification of genes and pathways regulated by pS727 that could be distinguished from those regulated by pY705 or by the combination of both. The identification of putative prognostic and predictive biomarkers that could be useful to determine the response to targeted therapies or eventually constitute novel therapeutic targets shall provide new tools to improve long-term outcomes in renal cancer patients.

## Results

### Characterization of STAT3 phosphomutants cell lines

**Figure 1:**
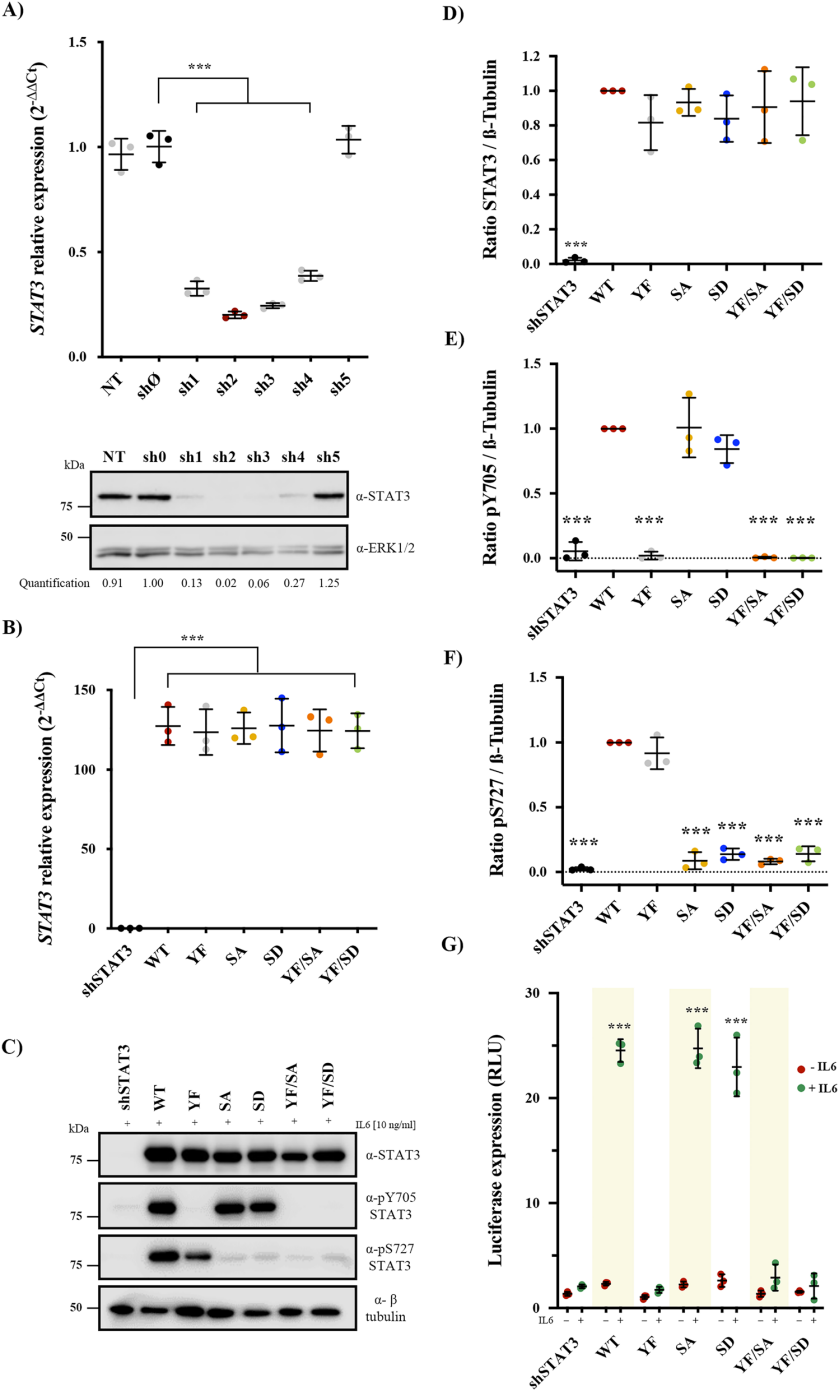
Generation of cellular models of STAT3 phosphomutants in 769-P cells. (**A**) Representative WB and RT-qPCR showing efficiency of 5 different shRNA against STAT3 after lentiviral transduction into human-derived ccRCC 769-P. (**B**) Relative expression levels of reintroduced STAT3 phosphomutants assessed by RT-qPCR. Data were normalized using TBP gene as the internal control. Quantification relative to shSTAT3 was performed by the 2-ddCt method. (**C**) Representative western blot showing protein levels of reintroduced STAT3 forms detected by antibodies against total STAT3 and the phosphorylated forms of Y705 and S727 residues. Treatment with 10 ng/ml IL6 for 30 min was used as classical activator of the canonical STAT3 pathway through pY705. Specific phospho-antibodies (pY705 and pS727) did not recognize mutations at these sites as indirect validation of proper substitutions. (**D**–**F**) Densitometric analysis of three independent western blots showing relative levels of total STAT3 (D), pY705 (E), and pS727 (F) normalized against β-tubulin (loading control). Data was compared to the wild-type form (WT). (**G**) Transcriptional activity of STAT3 phosphomutants was assessed in the absence or presence of 10 mg/ml of IL6, and measured as luciferase expression (relative light units, RLU). For all experiments, shSTAT3 corresponds to cells transduced with sh2 as well as overexpression empty-vector. Dots represent individual values and error bars indicate the mean ± SD, n = 3. Statistical significance: ***p < 0.001.

To explore the functional and transcriptional impact of STAT3 phosphorylation (via pY705 and/or pS727) in ccRCC, we generated novel ccRCC cell lines (769-P) carrying wild-type (WT) or phosphomutants forms of STAT3. First, 769-P cells were depleted of endogenous STAT3 using shRNA. From five different STAT3 shRNAs, sh2 had the higher knockdown efficiency at mRNA or protein levels, when compared to control cells (81% and 98% reduction, respectively) (Fig. 1A). Thus, sh2 cells were used in subsequent experiments.

Next, rescue STAT3 WT form (with synonymous mutations to skip shRNA2 recognized sequence) was expressed in STAT3 silenced cells (sh2) (Supplementary Fig. 1A). On this rescued STAT3 gene backbone, Y705 and S727 STAT3 phosphomutants were generated by introducing structurally similar amino acids that prevent (phosphoablative) or mimic (phosphomimetic) phosphorylation for each residue (Supplementary Fig. 1B): YF (p.Tyr705Phe), SA (p.Ser727Ala), SD (p.Ser727Asp), YF/SA (p.Tyr705Phe/p.Ser727Ala) and YF/SD (p.Tyr705Phe/p.Ser727Asp) (Table 1). A phosphomimetic substitution for Y705, however, was not possible since tyrosine is an aromatic amino acid and neither aspartic nor glutamic acid resembles the structure or charge density of a phosphotyrosine. To overcome this, we used interleukin-6 (IL6), a classic activator of the JAK/STAT3 pathway via pY705 that is elevated in the plasma of ccRCC patients^30^. IL6 stimulation at different concentrations and treatment times showed that 10 ng/ml for 15 min induced maximal Y705 phosphorylation (Supplementary Fig. 1C–E). No effects of IL6 stimulation were observed on S727 phosphorylation, which is constitutively phosphorylated in our experimental conditions.

**Table 1:** STAT3 phosphomutants.

STAT3	Phosphorylation state		Symbol
	Y705	S727	
WT	Phosphorylated by IL6	Phosphorylated	Y^P^/S^P^
YF	Phosphoablative	Phosphorylated	Y/S^P^
SA	Phosphorylated by IL6	Phosphoablative	Y^P^/S
SD	Phosphorylated by IL6	Phosphomimetic	Y^P^/S^Pm^
YF/SA	Phosphoablative	Phosphoablative	Y/S
YF/SD	Phosphoablative	Phosphomimetic	Y/S^Pm^

STAT3 mRNA levels were then tested by qPCR. Our results revealed similar mRNA levels in STAT3 WT and phosphomutants (YF, SA, SD, YF/SA, and YF/SD) (Fig. 1B). Expression of phosphomutants at protein level was confirmed by western blot, proving that all possible phosphorylation states for Y705 and S727 were obtained by specific amino acids substitution in combination with IL6 stimulation (Fig. 1C–F). To determine whether our STAT3 phosphomutants act through the canonical STAT3 activation pathway, we performed luciferase-reporter assays using STAT3 consensus promoter. Our data showed no significant transcriptional activity in any STAT3 phosphomutant in the absence of IL6. Importantly, IL6 promoted transcriptional activation of Y705 STAT3 forms (WT, SA, and SD) (Fig. 1G), indicating that STAT3 canonical pathway is pY705 dependent and pS727 independent.

The same approach and methodology were used to generate STAT3 phosphomutants in the 786-O cell line. As observed in the 769-P cells, STAT3 phosphomutants expression in the 786-O cell line exhibited different phosphorylation levels for Y705 and S727 (YF, SA, and SD) upon specific amino acids substitution in combination with IL6 stimulation (Supplementary Fig. 2A).

### STAT3 overexpression promotes a tumoral phenotype in ccRCC

Next, we studied the impact of STAT3 WT overexpression on transformation processes in ccRCC by analyzing different oncogenic traits including cell viability, migration, clonogenicity and anchorage-independent growth. All experiments were carried out in the presence of 10 ng/ml IL6. Thus, STAT3 WT had both Y705 and S727 residues phosphorylated (Y^p^/S^p^).

**Figure 2:**
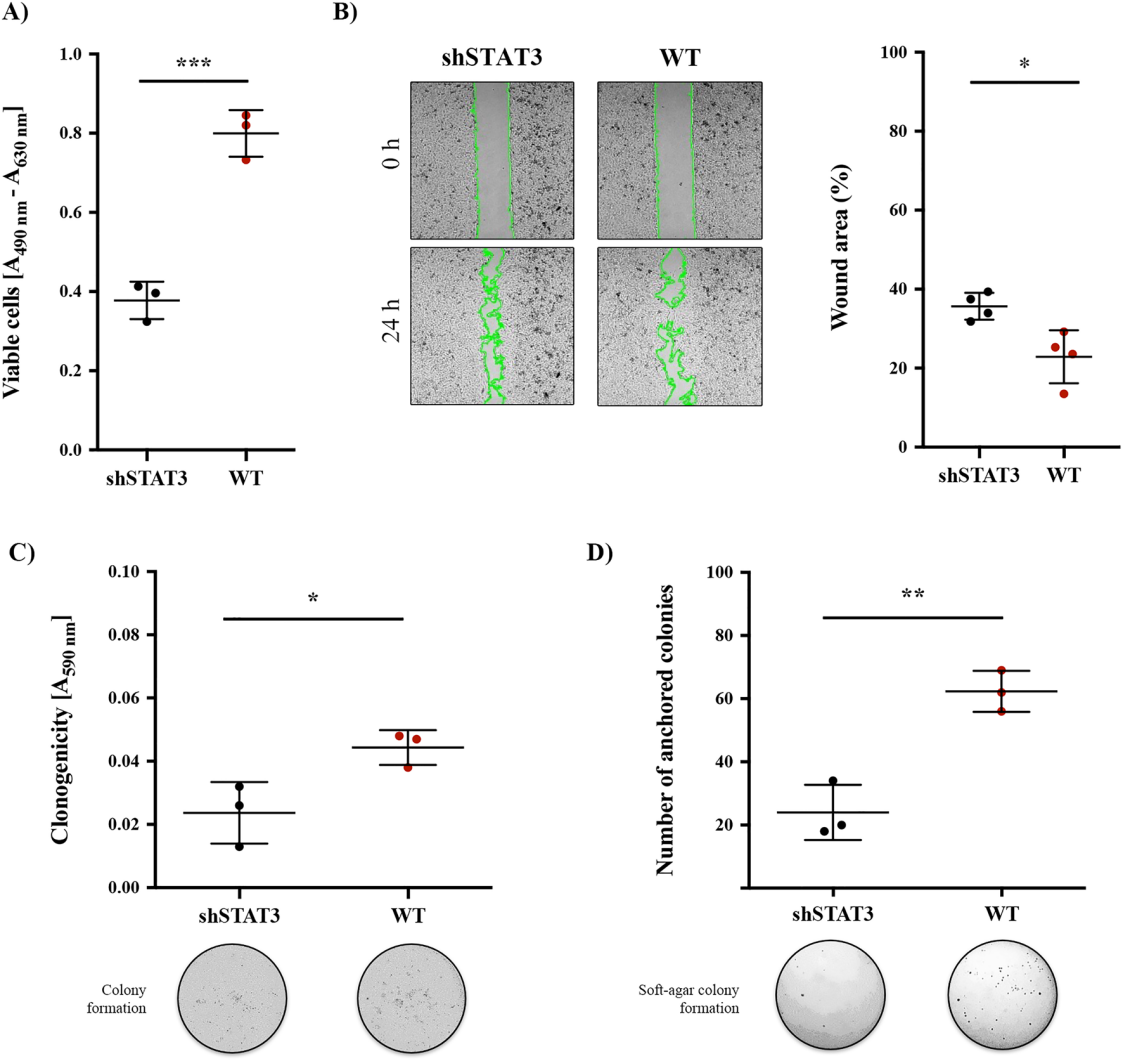
STAT3 overexpression promotes a tumoral phenotype in 769-P cells. (**A**) Cell proliferation was calculated 72 h after seeding. (**B**) Representative pictures of wound-healing assays to evaluate cell migration. Wound closure was analyzed, and pictures were taken at 0 and 24 h. Limits of the wound were underlined in green. The percentage of the total wound area to be closed was calculated using ImageJ software. (**C**) Clonogenic capacity was evaluated by crystal violet staining after 7 days. Colony heterogeneity is shown in representative photographs. (**D**) Soft agar assay was performed to determine the anchorage-independent growth after 28 days. Colony formation is shown in representative photographs. All experiments were carried in the presence of 10 ng/ml IL6. Dots represent individual values and error bars indicate the mean ± SD, n = 3. Statistical significance: *p < 0.05, **p < 0.01, ***p < 0.001.

Our results showed that STAT3 WT overexpression induced a twofold increase in the number of viable cells compared to shSTAT3 cells (*p* < 0.001) in the 769-P cell line (Fig. 2A) and in the 786-O cell line when compared to shSTAT3 (Supplementary Fig. 2B). Cell motility was evaluated by wound healing assays in the absence of FBS to ensure that migration rather than proliferation was considered. Our data showed that STAT3 WT cells covered a higher area of the wound after 24 h than shSTAT3 cells (13% higher, *p* < 0.05) in the 769-P cells (Fig. 2B). This feature was also observed in the 786-O cell line (Supplementary Fig. 2C). Similarly, an increased colony formation capacity was found for STAT3 WT cells compared to shSTAT3 cells (1.8 fold increase, *p* < 0.01), in alignment with a more aggressive phenotype (Fig. 2C). Finally, the anchorage-independent growth assay demonstrated that STAT3 WT confers the ability to create cell niches and independent growth in a solid surface compared to shSTAT3 cells (2.6-fold increase, *p* < 0.001) (Fig. 2D). Overall, our results indicate that Y705/S727 phosphorylated STAT3 WT overexpression promotes a ccRCC tumoral phenotype.

### STAT3 phosphorylation at S727 aggravates ccRCC

**Figure 3:**
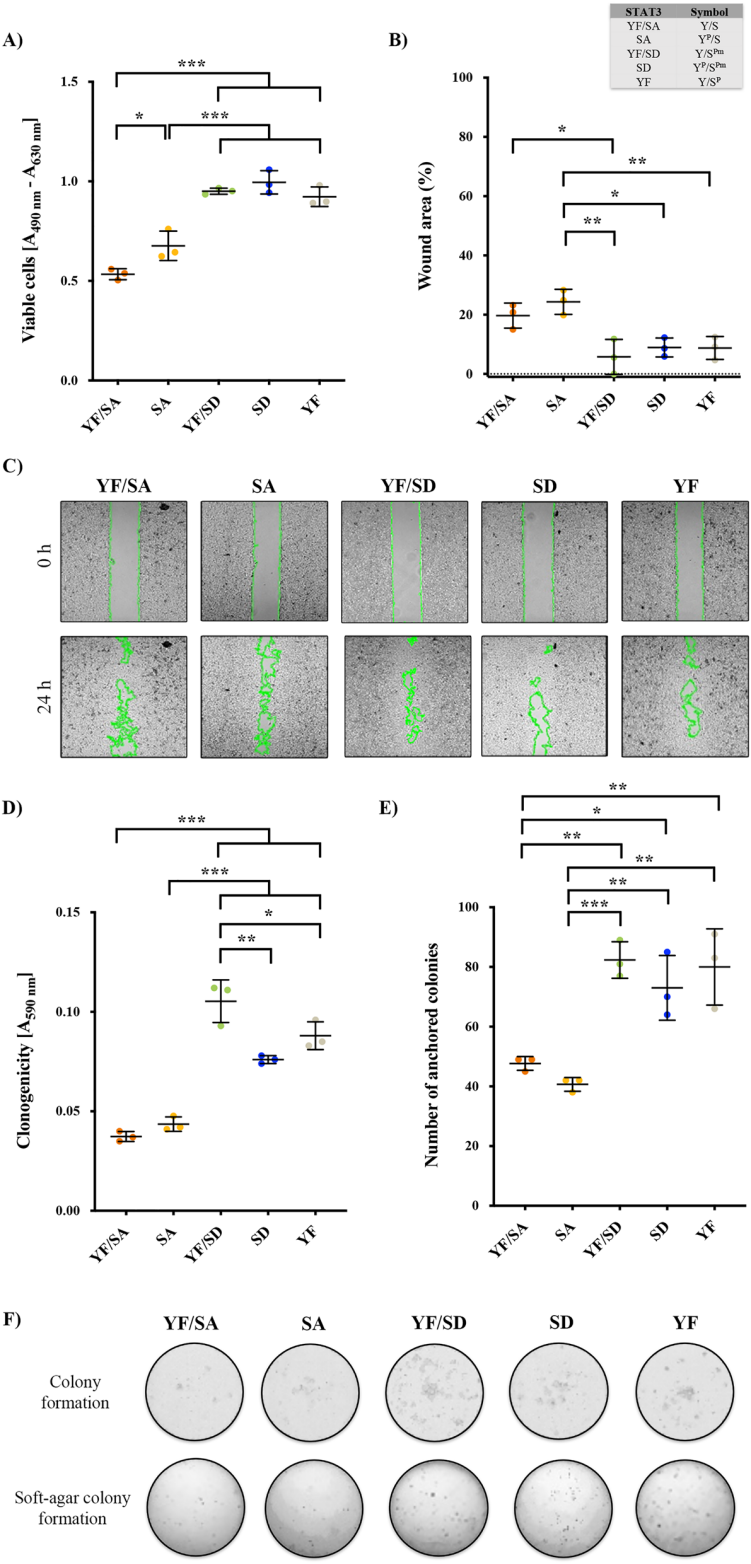
Phosphorylation of S727 aggravates the tumoral phenotype in 769-P cells. (**A**) Proliferation capacity was evaluated at 72 h by subtracting the absorbance of each sample at 630 nm (reference wavelength background) from the absorbance at 490 nm. (**B**) Cell migration was evaluated by wound-healing assay. Wound closure was observed, and pictures were taken at 0 and 24 h. The percentage of the total wound area to be closed was calculated using ImageJ software. **C)** Representative images of wound closure at 0 and 24 h. For better observation of the healing, limits of the wound were underlined in green. (**D**) Clonogenic capacity was evaluated by crystal violet staining after 7 days. (**E**) Soft agar assay was performed to determine the anchorage-independent growth after 28 days. (**F**) Representative images of colony formation and soft-agar colony formation for all STAT3 mutant cell lines. All experiments were carried on in the presence of 10 ng/ml IL6. Dots represent individual values and error bars indicate the mean ± SD, n = 3. Statistical significance: *p < 0.05, **p < 0.01, and ***p < 0.001.

Once determined the effects of STAT3 WT overexpression, we functionally characterized STAT3 phosphomutants cell models. Our results showed that phosphomimetic S727 mutants SD (Y^P^/S^Pm^) and YF/SD (Y/S^Pm^), as well as the YF mutant (Y/S^P^) which is endogenously S727 phosphorylated, exhibited higher cell viability rate (1.9-, 1.8- and 1.7-fold increase, respectively; *p* < 0.001) (Fig. 3A), increased wound healing (10.8%, 13.9% and 10.9% higher, respectively; *p* < 0.05) (Fig. 3B,C), enhanced clonogenicity (2.7-, 3.3- and threefold increase, respectively; *p* < 0.001) (Fig. 3D, F), and higher anchorage-independent growth (1.5-, 1.7- and 1.7-fold increase, respectively; *p* < 0.01) (Fig. 3E, F) than unphosphorylated STAT3 mutant (YF/SA, Y/S). Importantly, SD mutant (Y^P^/S^Pm^) exhibited a significantly increased performance in all features when compared to SA mutant (Y^P^/S), pointing to pS727 as the main responsible residue for STAT3 oncogenic outcome. It is also important to pinpoint that in the absence of pY705 no significant changes were observed between phosphomimetic or endogenously phosphorylated S727 residue regarding tumoral phenotype. In summary, pS727 exerts the strongest effect on STAT3 tumoral behavior, which agrees with a more aggressive ccRCC phenotype in patients.

In agreement with 769-P STAT3 phosphomutants phenotype, SA, SD, and YF mutants in 786-O cells exhibited significant differences in their proliferation capacity being greater in YF (Y/S^P^) and SD (Y^P^/S^Pm^) than in the SA mutant (Y^P^/S) (Supplementary Fig. 2D). Moreover, the SA mutant (Y^P^/S) was the one with the lowest migration capacity (Supplementary Fig. 2E).

### STAT3 phosphorylation at S727 enhances transcriptional activity

**Figure 4:**
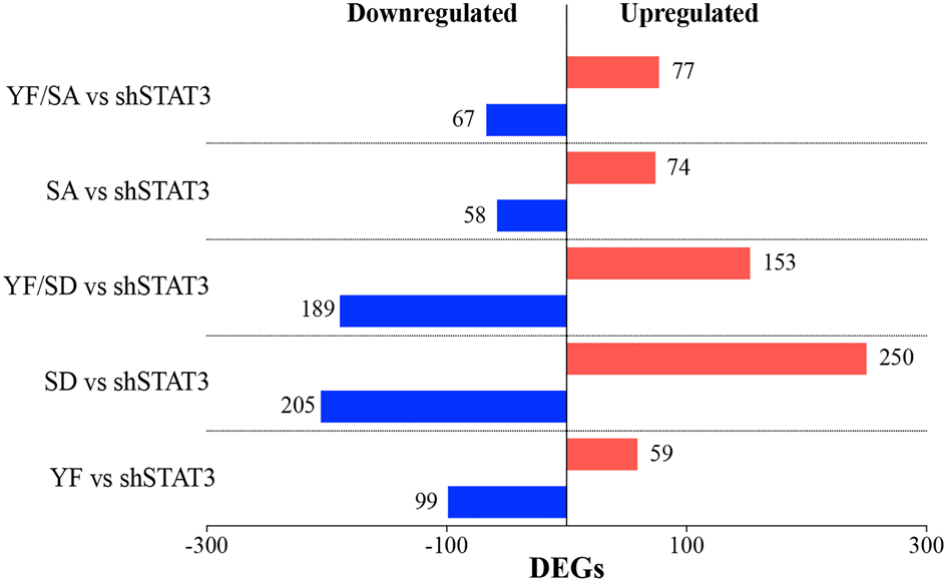
Differentially expressed genes in each STAT3 phosphomutant. Selection of differentially expressed genes (DEGs) based on a FDR < 0.25 and a logFC >|0.5|.

In order to identify genes and pathways regulated by different STAT3 phosphorylation states, we evaluated STAT3 phosphomutants’ effect on global gene expression. Microarray results confirmed that STAT3 mutants’ expression levels were alike regarding log2 fold-change (logFC) and adjusted *p*-value (adj.P. Value) (Supplementary Table 1), allowing an unbiased comparison of transcriptional activity among them. For comparative analysis of microarray data, differentially expressed genes (DEGs) were defined as those presenting an FDR < 0.25 and a logFC >|0.5|. Up- and down-regulated genes in STAT3 phosphomutants compared to shSTAT3 cells are shown in Fig. 4. We found that total number of up- and down-regulated genes between mutant YF/SA (Y/S, no phosphorylated) and SA (Y^P^/S, Y705 phosphorylated) is similar (144 vs. 132 genes, respectively). Endogenous S727 phosphorylation (YF, Y/S^P^) changes 158 genes’ expression, while phosphomimetic form (YF/SD, Y/S^Pm^) increases STAT3-regulated genes to 342, achieving the highest transcriptional activity when both residues (SD, Y^P^/S^Pm^) are phosphorylated (n = 455) (Fig. 4).

To validate the results obtained from the microarray, a selection of 4 of the most differentially expressed genes among initial comparisons were selected including *KRT20*, *FN1*, *ALPK2*, and *ANKS4B,* in 769-P cell line (Supplementary Figure 3).

RT-qPCR results presented in Supplementary Fig. 3 agree with microarray results shown in Supplementary Table 2. The impact of STAT3 phosphomutants in selected target genes was also studied in another ccRCC-derived cell line (786-O), which showed a similar gene expression pattern to that found in 769-P (Supplementary Fig. 4).

**Figure 5:**
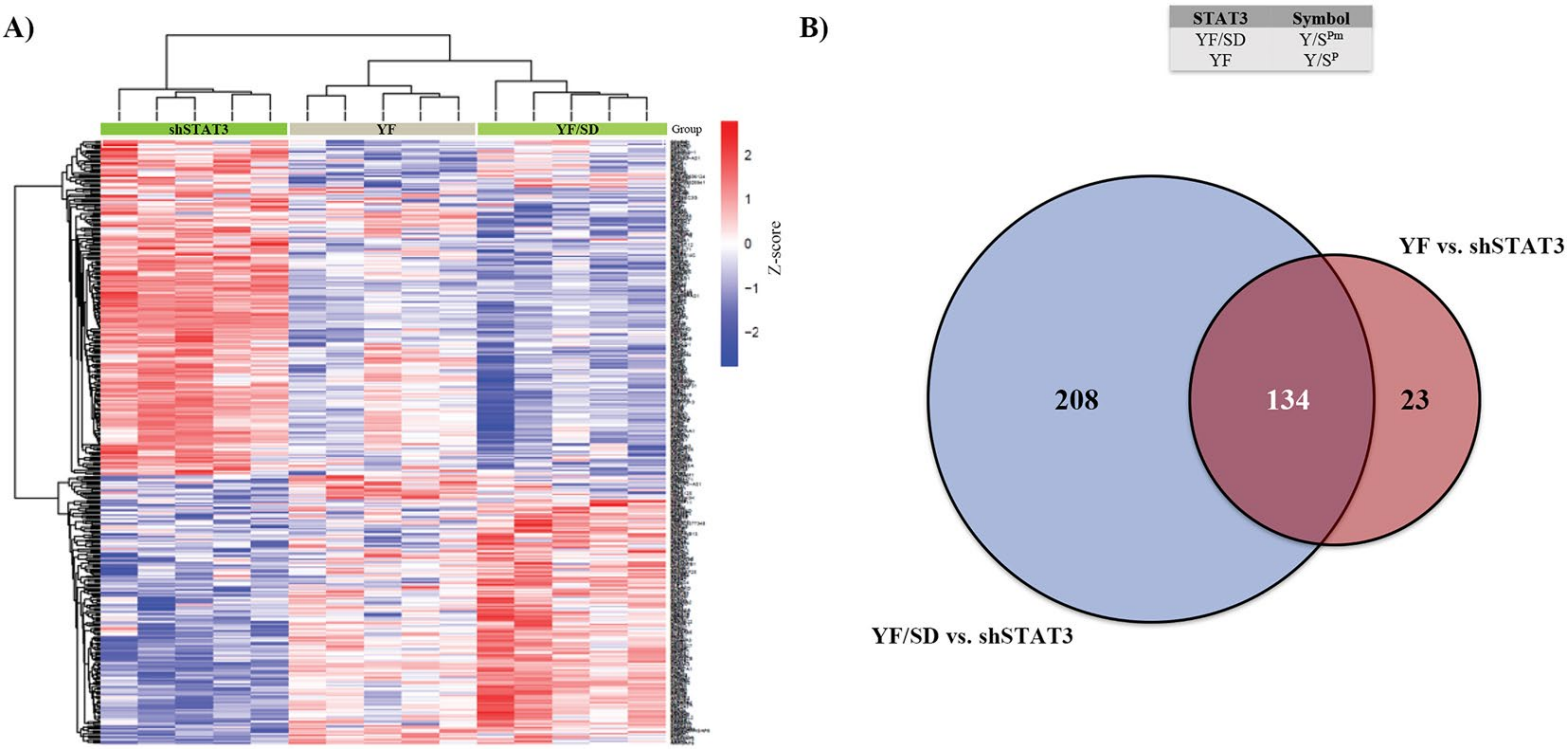
Phosphomimetic mutation SD mimics native S727 phosphorylation. (**A**) Visualization of common expression patterns (heatmap) after cluster analysis of genes showing differential expression (FDR < 0.25 and a logFC >|0.5|). Expression values for each gene (row) are normalized across all samples (columns) by Z-score. Both column and row clustering were applied, and distinct gene clusters identified are shown to illustrate the major expression patterns observed in the data. (**B**) Venn diagram of upregulated and downregulated genes in YF versus shSTAT3 and YF/SD versus shSTAT3 reveals that they share 134 genes corresponding to 84.8% of the total expression of YF mutant.

Results from functional assays and visualization of common expression patterns between YF/SD (Y/S^Pm^) and YF (Y/S^P^) indicate that the hierarchical clustering of DEGs in both mutants is similar (Fig. 5A), which is not surprising since they represent the same STAT3 phosphorylation state (unphosphorylated Y705 and phosphorylated S727). Therefore, YF mutant (Y/S^P^) represents a proof of concept of the S727 phosphomimetic mutation SD. Our results also showed that 134 (84.8%) of DEGs found in YF (Y/S^P^) were differentially expressed in YF/SD (Y/S^Pm^) (Fig. 5B). These results suggest that, although endogenous pS727 does not reach the transcriptional strength of the S727 phosphomimetic mutation (SD), it maintains the expression of gene sets necessary to produce a similar phenotypic oncogenic outcome as shown in our cell culture assays. Based on these results and for experimental clarity, only the YF/SD (Y/S^Pm^) mutant was used for subsequent comparisons.

**Figure 6:**
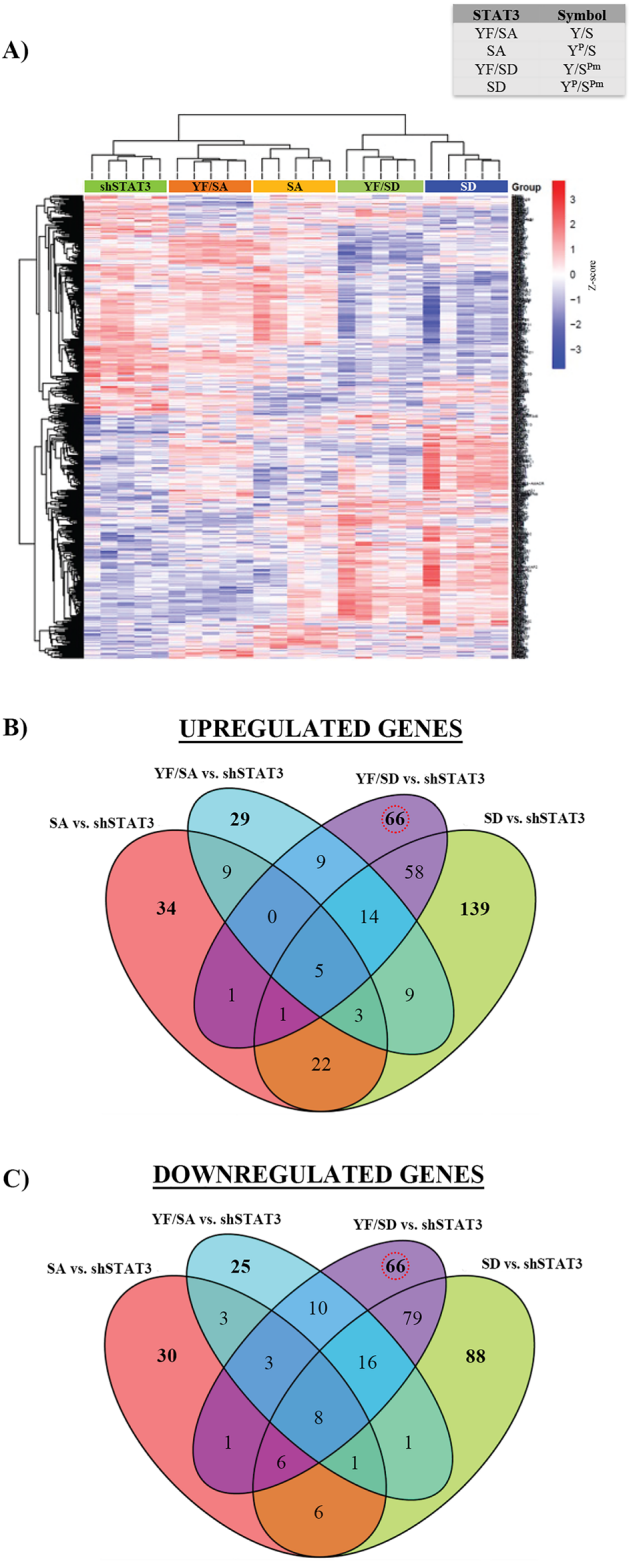
Phosphorylation of S727 exerts a more forceful effect on STAT3 activation than phosphorylation of Y705. (**A**) Visualization of common expression patterns (heatmap) after cluster analysis of genes showing differential expression (FDR < 0.25 and a logFC >|0.5|) for all STAT3 phosphomutants (YF/SA, SA, YF/SD, and SD) compared to the absence of STAT3 (shSTAT3). Expression values for each gene (row) are normalized across all samples (columns) by Z-score. Both column and row clustering were applied, and distinct gene clusters identified are shown to illustrate the major expression patterns observed in the data. Venn diagrams of (**B**) upregulated and (**C**) downregulated genes in multiple comparisons where it can be observed common genes at the intersections and specific genes of each phosphorylation state at the outer sets (bold numbers). STAT3 phosphorylation states YF/SD and SD (where S727 is phosphorylated) are the ones with the highest number of specifically DEGs. In red circles are highlighted the genes specifically dependent on pS727 and independent of pY705.

Analysis of differential gene expression allowed the determination of specific lists of DEGs for each STAT3 phosphorylation state. The top 30 DEGs (15 up- and 15 down- regulated) are shown in Tables 2, 3, 4and5. Multiple comparisons of STAT3 phosphomutants were performed using the following conditions: YF/SA versus shSTAT3, SA versus shSTAT3, YF/SD versus shSTAT3, and SD versus shSTAT3. The resultant heatmap shows a gradual transition of STAT3 transcriptional activity as both residues become phosphorylated (Fig. 6A). Unphosphorylated STAT3 (YF/SA, Y/S) is the closest mutant to STAT3 depletion (shSTAT3), followed by Y705 phosphorylation (SA, Y^P^/S). This expression pattern begins to be the opposite when S727 becomes phosphorylated (YF/SD, Y/S^Pm^) and, finally, phosphorylation of both residues (SD, Y^P^/S^Pm^) leads to a complete reversed expression pattern. Venn diagrams of up- and down- regulated DEGs allowed the visualization of common and specific genes regulated by different STAT3 phosphomutants. This analysis revealed that S727 phosphomimetic mutants (YF/SD -Y/S^Pm^- and SD -Y^P^/S^Pm^-) specifically regulate a higher number of DEGs compared to S727 phosphoablative mutants (YF/SA, Y/S, and SA) (Fig. 6B,C). Altogether, these results demonstrate that pS727 exerts a more powerful effect on STAT3-mediated gene transcription than pY705. In more detail, S727 phosphorylation specifically regulated 132 DEGs (66 up- and 66 down-regulated) that were present in YF/SD mutant but not in any other STAT3 phosphomutant (Fig. 6B,C) (Supplementary Table 3).

**Table 2:** 30 most DEGs regulated by unphosphorylated STAT3 (YF/SA vs. shSTAT3).

Upregulated genes			Downregulated genes		
Gene symbol	logFC	FDR	Gene symbol	logFC	FDR
CNTN6	1.6522	8.67E−10	C1S	− 2.4756	1.80E−14
WNT7A	1.1641	4.44E−05	CFH	− 2.4062	9.42E−13
PLPPR5	1.0606	1.35E−04	TRIM22	− 1.6938	1.04E−09
RENBP	0.9980	3.80E−04	CFB	− 1.6682	5.38E−09
EPHA7	0.9218	8.41E−06	CFI	− 1.3759	3.56E−05
HS3ST3B1	0.9094	8.97E−06	CP	− 1.3139	5.38E−09
NPNT	0.8948	2.72E−03	LCP1	− 1.1433	3.80E−04
HS3ST3A1	0.8687	4.35E−05	GBP1	− 0.9470	2.40E−03
DOCK2	0.8625	3.77E−03	IRF9	− 0.9241	2.17E−05
SLC6A13	0.8311	7.48E−02	TGM2	− 0.9141	8.97E−06
PSG8	0.8226	4.64E−03	GBP2	− 0.9089	2.10E−06
PSG1	0.8208	3.58E−04	EPAS1	− 0.8568	2.45E−06
AKR1B10	0.8163	4.64E−03	APOL1	− 0.8358	8.97E−06
PSG3	0.8143	2.64E−03	CST2	− 0.8335	2.24E−03
AKR1B15	0.8124	2.43E−04	RAB27B	− 0.8082	5.48E−05

**Table 3:** 30 most DEGs regulated by Y705 phosphorylation (SA vs. shSTAT3).

Upregulated genes			Downregulated genes		
Gene symbol	logFC	FDR	Gene symbol	logFC	FDR
NNMT	1.3285	1.06E−06	LCP1	− 2.0427	1.73E−09
SAA1	1.0696	1.50E−03	ANKS4B	− 1.1794	2.17E−03
FGB	1.0432	9.21E−07	IL36B	− 1.1120	1.82E−02
KCNIP1	1.0005	2.51E−05	IL18	− 1.0888	1.46E−06
AGT	0.9857	2.50E−03	UBD	− 0.9863	1.50E−03
CNTN6	0.9215	8.74E−04	GPR34	− 0.9197	1.54E−02
SCN9A	0.9091	5.39E−04	GALM	− 0.9078	1.02E−02
TRMT9B	0.8953	4.44E−03	PDE9A	− 0.8935	5.39E−04
CGB2	0.8951	2.81E−03	NOS3	− 0.8700	4.63E−03
PTGDS	0.8490	1.80E−02	SLC6A20	− 0.8328	1.09E−02
TCP11L2	0.8231	4.14E−02	TRIM31	− 0.8317	2.19E−02
CDH6	0.8186	8.74E−04	C1S	− 0.8236	7.74E−03
EFCAB13	0.8172	4.14E−02	GUCA2A	− 0.8108	1.38E−02
CGB7	0.8121	7.74E−03	SLCO1B3	− 0.7957	3.62E−02
TOMM20L	0.7907	2.30E−02	GFPT2	− 0.7916	1.54E−02

**Table 4:** 30 most DEGs regulated by S727 phosphorylation (YF/SD vs. shSTAT3).

Upregulated genes			Downregulated genes		
Gene symbol	logFC	FDR	Gene symbol	logFC	FDR
CNTN6	1.8954	5.22E−12	LCP1	− 3.1203	4.90E−16
PLPPR5	1.6667	2.66E−09	CFH	− 2.6428	2.31E−14
A1CF	1.5088	4.13E−08	C1S	− 2.5312	5.19E−15
ANKS4B	1.4402	2.25E−05	TRIM22	− 2.1121	6.18E−13
CLRN3	1.3499	1.26E−04	CFB	− 1.8762	1.04E−10
CLTRN	1.3159	9.60E−06	IGFBP3	− 1.7921	3.47E−12
ERBB3	1.2305	7.23E−04	SLCO1B3	− 1.4707	2.13E−06
NR0B2	1.2278	1.97E−07	GBP1	− 1.4539	3.99E−07
CIDEB	1.2200	2.79E−05	TNFAIP3	− 1.3545	3.46E−04
MYO7B	1.2044	3.63E−05	CLEC4E	− 1.3444	2.80E−04
GLUL	1.1894	7.71E−09	EDN1	− 1.3386	8.50E−05
ACE2	1.1766	1.82E−04	FN1	− 1.3356	1.89E−09
METTL7A	1.1744	3.23E−03	MYL9	− 1.2956	1.25E−06
KCNJ3	1.0780	5.91E−04	SNAI2	− 1.2905	2.12E−03
UGT2A3	1.0604	8.98E−06	EDN2	− 1.2812	1.16E−04

**Table 5:** 30 most DEGs regulated by both Y705 and S727 phosphorylation (SD vs. shSTAT3).

Upregulated genes			Downregulated genes		
Gene symbol	logFC	FDR	Gene symbol	logFC	FDR
CNTN6	2.7715	1.22E−17	LCP1	− 3.1181	3.36E−16
PLPPR5	1.6484	6.19E−09	PTX3	− 1.7359	1.83E−04
SLC6A13	1.4414	3.33E−05	GBP1	− 1.6894	1.57E−08
A1CF	1.3771	5.03E−07	EDN2	− 1.6772	1.01E−06
SLCO4C1	1.2966	2.25E−05	TRIM22	− 1.5995	6.17E−09
FGB	1.2790	1.80E−09	CLEC4E	− 1.5649	2.41E−05
DDIT4	1.2570	5.03E−07	CTSS	− 1.4597	2.57E−06
C1QTNF3-AMACR	1.2482	3.06E−06	UBD	− 1.3528	1.85E−06
TEAD2	1.2189	1.85E−05	TNFAIP3	− 1.3526	3.40E−04
AMACR	1.2057	1.42E−05	ROR1	− 1.3444	3.44E−07
PLXDC2	1.1949	2.71E−07	TAGLN	− 1.3269	1.70E−03
KCNJ13	1.1894	1.49E−03	ANKRD1	− 1.3062	1.34E−03
ANKS4B	1.1861	4.17E−04	MYL9	− 1.2977	1.71E−06
METTL7A	1.1761	2.62E−03	C1S	− 1.2825	2.38E−06
CFI	1.0827	6.11E−04	SNAI2	− 1.2576	2.38E−03

Specific DEGs on SD (Y^P^/S^Pm^) mutant are representative of STAT3 activation via both pY705 and pS727 and correlate with the maximal STAT3 phosphorylation state that might be found in ccRCC patients^28,29^. When both residues are phosphorylated, we obtained 227 specific DEGs, 139 of them upregulated and 88 downregulated (Supplementary Table 4). Additionally, Venn diagrams also showed 265 common genes (131 up- and 134 down- regulated) scattered among all STAT3 phosphomutants. These genes (located at the intersections of Venn diagrams) do not follow a clear pattern regarding pY705 or pS727, suggesting that despite being STAT3 regulated they do not respond to the phosphorylation state and might be controlled by other post-translational modifications (Fig. 6B,C).

### pS727-dependent genes are linked to hallmarks of cancer development

**Figure 7:**
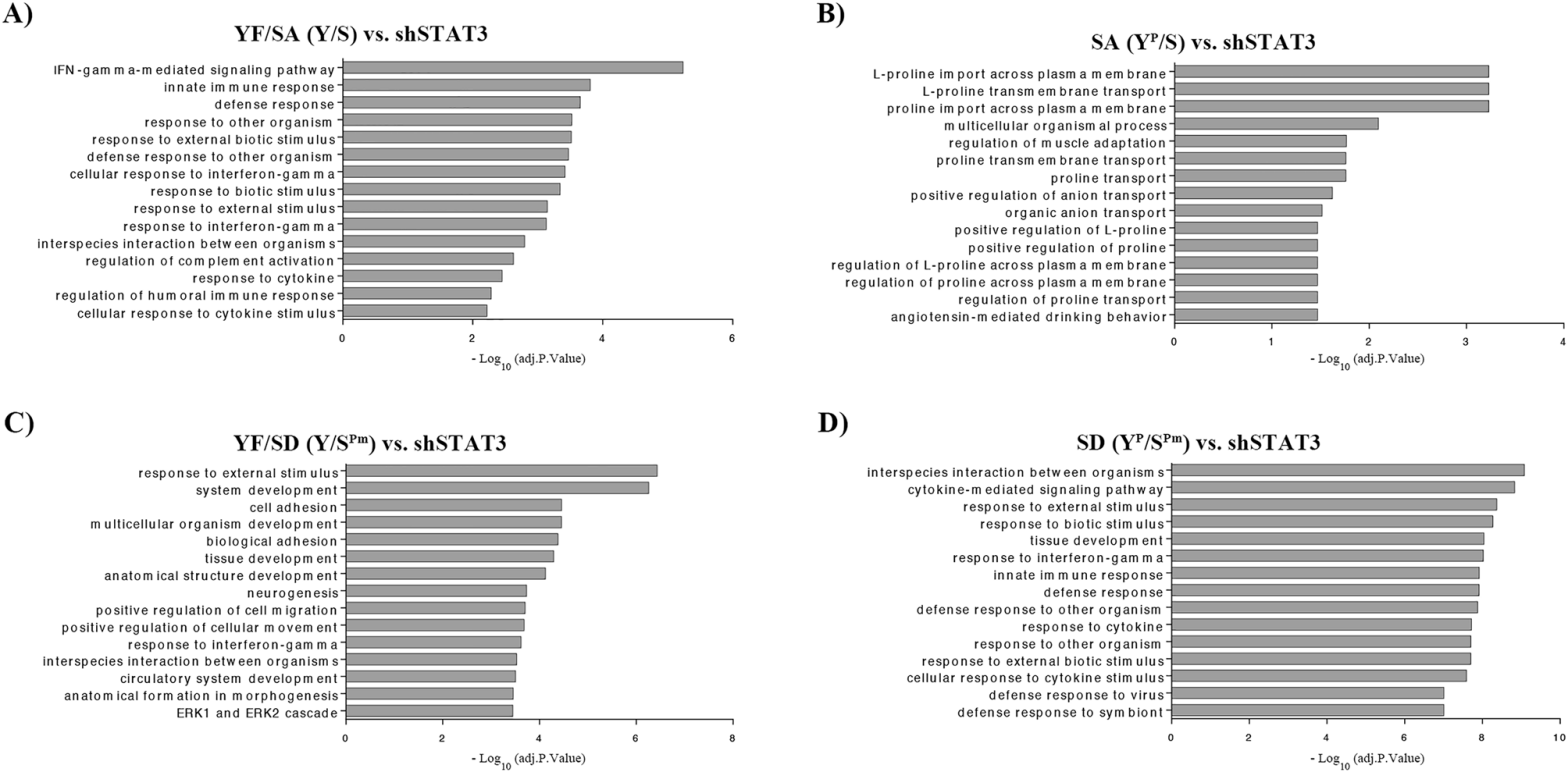
Biological processes related to different STAT3 phosphorylation states. The top 15 overrepresented Gene Ontology terms obtained from g:Profiler analysis for comparisons: (**A**) YF/SA versus shSTAT3, (**B**) SA versus shSTAT3, (**C**) YF/SD versus shSTAT3, and (**D**) SD versus shSTAT3. The analysis was performed using filtered (FDR < 0.25 and a logFC >|0.5|) and ranked lists (according to FDR) of differentially expressed genes from microarray analysis.

The biological significance of specific DEGs for different STAT3 phosphomutants was determined by the identification of their associated signaling pathways. The top 15 statistically significant overrepresented terms are shown in Fig. 7. YF/SA mutant (Y/S) yielded a list of 144 DEGs that were related to a total of 27 biological processes, most of them linked to interferon-gamma (IFNγ) and defense responses (Fig. 7A). The transcriptional activity of the SA mutant (Y^P^/S) regulates the expression of 132 genes. This phosphorylation state exhibited the lowest number of DEGs which were linked to 18 enriched terms of which the majority have implications on proline transport (Fig. 7B). YF/SD mutant (Y/S^Pm^) by itself regulates the expression of 342 genes that are significantly associated with 96 biological processes such as cell adhesion, proliferation and development, cell migration, angiogenesis, differentiation, response to stress, and response to decreased oxygen levels, all considered hallmarks of cancer^31^ (Fig. 7C). This outcome indicates that pS727 could have an important role in oncogenesis and reinforces our results from in vitro experiments.

**Figure 8:**
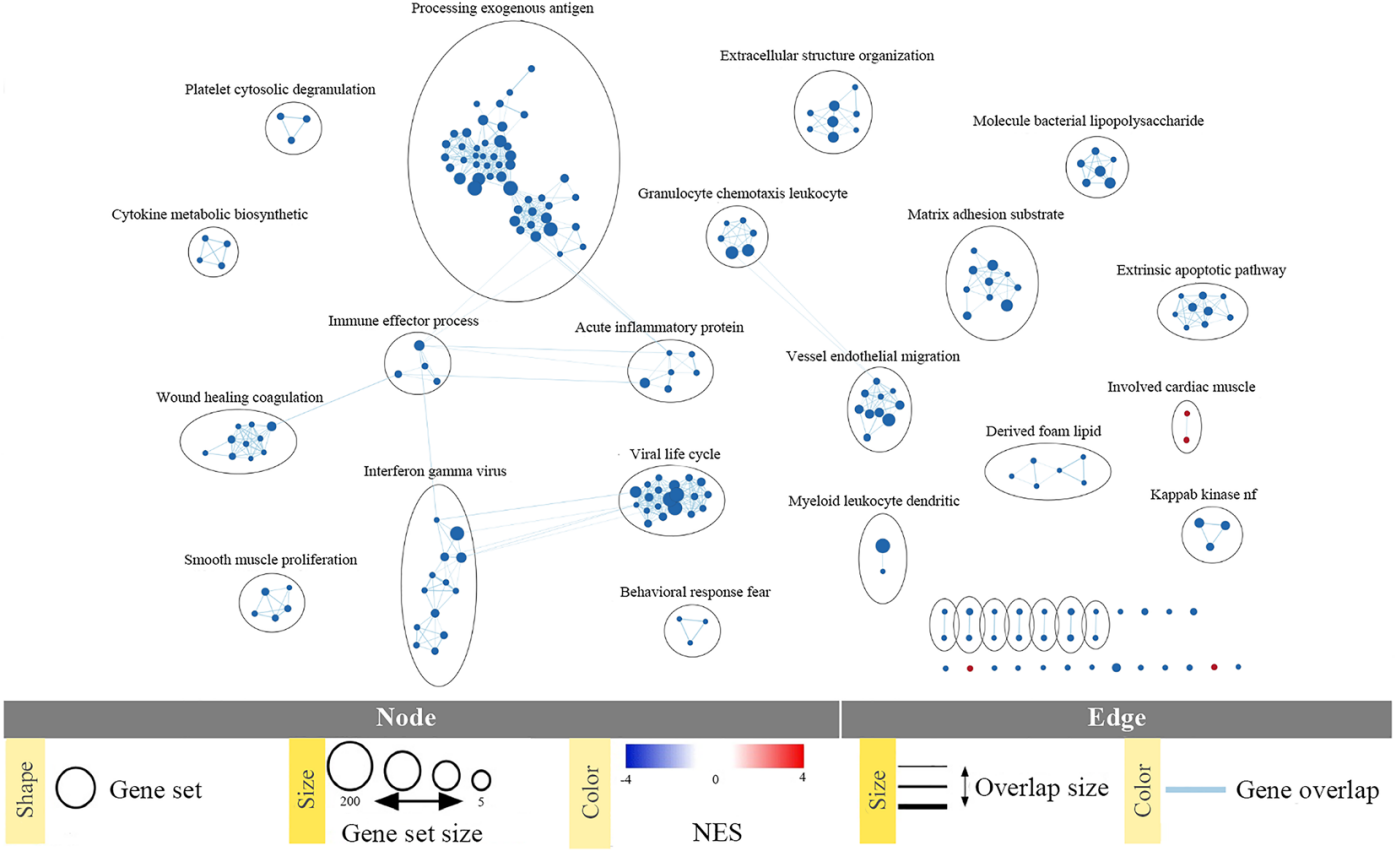
Molecular pathways where pS727-dependent genes are involved. Gene Set Enrichment Analysis (GSEA) was performed, and results visualized with Enrichment Map plugin for Cytoscape software setting thresholds for *p* value at < 0.01 and for FDR at < 0.1. Node size corresponds to the number of genes comprising the gene set. Normalized enrichment scores (NES) for gene set are represented by node’s color (red = upregulation, blue = downregulation). To identify redundancies between gene sets, the nodes are connected with edges if their contents overlap by more than 50%. Thickness of the edge corresponds to the size of the overlap.

Finally, SD mutant (Y^P^/S^Pm^) presented the highest number of DEGs (455) and the highest number of biologically significant terms (152), which correlates with previous reports indicating that phosphorylation at both sites produces an optimal STAT3 activation^30,31^. Genes controlled by this phosphorylation state are related to oncogenic processes—similar to YF/SD mutant (Y/S^Pm^)—along with others responsible for external stimuli response. Defense responses, cell differentiation, migration, angiogenesis, proliferation, adhesion, apoptosis, extracellular matrix organization, wound healing, cell communication, and regulation of proline transport are some of them (Fig. 7D). The activation of these signaling pathways reveals the striking effect of pS727 on STAT3-mediated biological significance by accentuating the tumoral phenotype over the effects of pY705 in ccRCC. To correlate the specific DEGs regulated by pS727 within the whole biological context of YF/SD (Y/S^Pm^), we identified the molecular pathways where those 132 pS727-dependent genes were involved. The analysis showed that pS727-dependent genes mainly contribute to the downregulation of immune response and apoptosis inhibition. Regulation of cell proliferation, adhesion, migration, and angiogenesis were also found related to S727 phosphorylation (Fig. 8).

## Discussion

In this work, we have generated novel ccRCC-derived cell lines to analyze the effects produced by STAT3 silencing and the impact caused by Y705 and S727 phosphorylation in cancer hallmarks such as proliferation, migration, colony formation, and anchorage-independent growth. In addition, we have identified genes and pathways differentially expressed in cell lines overexpressing STAT3 WT or the different STAT3 phosphomutants.

Although STAT3 oncogenic properties were classically attributed to Y705 phosphorylation, S727 phosphorylation has also emerged as an event that enhances STAT3 transcriptional activity in addition to non-genomic roles that promote cancer development^26,27,32,33^. Nevertheless, many studies on the role of pS727 have been done under the presumption that pY705 occurs prior to pS727, underestimating the role of pS727 by itself. To counter this, some authors have recently shown that STAT3 can be activated by pS727 in the absence of pY705 in non-cancerous and cancerous cells^17,34^. Our experimental design was intended to avoid the dynamic modulation of STAT3 phosphorylation based on the premise that phosphorylation at one residue could impact the phosphorylation state in the other^26^. Accordingly, we produced a collection of ccRCC-derived cell lines carrying STAT3 phosphomutants that cover all possible phosphorylation combinations between the Y705 and S727 residues to clearly assess their role. Although this approach may not faithfully represent all possible STAT3 phosphorylation state levels exhibited by tumors^28,29^, it intends to define the contribution of pY705 and pS727 in ccRCC biology at the functional and molecular levels.

Initially, and according to published reports, we considered the use of aspartic or glutamic acid to mimic Y705 phosphorylation^35–38^, however, we did not find an impact on overall gene expression assays with the aspartic phosphomimetic form (not shown). To overcome this problem and given that IL6 is the gold-standard activator of STAT3 via pY705^39^ and ccRCC patients exhibit sustained high IL6 plasma levels^30^ we treated the cells with IL6 for Y705 phosphorylation. Although there are studies showing that IL6 can also stimulate S727 phosphorylation by an ERK-independent process^26,40–43^, we did not observe this effect in our cell lines where S727 is constitutively phosphorylated through IL6-independent mechanisms. Regarding S727, we produced a phosphomimetic substitution with aspartic acid (SD). Comparison of YF/SD (Y/S^Pm^) with YF (Y/S^P^), revealed no major changes in phenotypic and gene expression patterns except by the fact that phosphomimetic mutation SD exhibited higher transcriptional potency, regulating a larger number of DEGs than the endogenously phosphorylated S727. Hence, we conclude that the S727 phosphomimetic mutation (SD) is biologically functional and that the results obtained with this mutant are reliable.

STAT3 can be activated by two different main pathways, the canonical pathway with nuclear transcription of target genes, and the non-canonical pathway which includes genomic and non-genomic STAT3 functions. Luciferase-reporter assay with the STAT3 consensus sequence promoter revealed that only mutants with phosphorylated Y705 by IL6 (SA, Y^P^/S and SD, Y^P^/S^Pm^) exhibited transcriptional activity, indicating that pY705 is a key event for STAT3 functioning through the canonical pathway and, importantly, that this is independent of S727 phosphorylation state. Therefore, the effects on gene expression exhibited by S727 phosphomimetic mutants (SD) are not mediated by the STAT3 consensus element. Although we have not explored the mechanisms to explain these effects, previous reports have described that unphosphorylated STAT3 (which in the literature refers only to the absence of pY705) regulates transcription of a subclass of target genes by forming a complex with the nuclear factor kappa B (NFĸB), as a non-canonical STAT3 activation^44,45^.

Results obtained in our study indicate that STAT3 WT (Y^p^/S^p^) significantly sustains the tumoral phenotype when compared to shSTAT3 cells. Moreover, functional characterization of STAT3 phosphomutant cell lines has shown that S727 phosphomimetic forms (YF/SD, Y/S^Pm^ and SD, Y^P^/S^Pm^) significantly stood out in all oncogenic traits studied, independently of Y705 phosphorylation state. Different from STAT3 phosphomutants’ general behavior, proliferation analysis showed significant differences between the number of viable cells at 72 h in SA mutant (Y^P^/S) compared to unphosphorylated STAT3 (YF/SA,Y/S). Similarly, colony formation assays showed that SD mutant (Y^P^/S^Pm^) formed significantly fewer colonies than YF/SD mutant (Y/S^Pm^). Since both experiments evaluate the cell ability to thrive under different situations, these results suggest that when cells are nearby (proliferation assay), pY705 by itself can significantly contribute to cell growth; however, in the absence of cell–cell interactions (clonogenic assay), cellular division is mainly promoted by pS727.

In agreement with our results, other authors have also shown that, in the absence of pY705, pS727 activates STAT3 signaling and promotes enhancing survival in macrophages, neuronal stem cells, and prostate cancer cells^17,46^. Furthermore, a constitutively active form of STAT3 (STAT3C)—consisting of a dimerizing STAT3 without the requirement of pY705—showed that upon transfection, STAT3 was phosphorylated at S727 but not at Y705. Overexpression of STAT3C could drive transcription, induce transformation and enhance tumorigenicity in immortalized fibroblast and epithelial cells indicating that pS727 was indispensable for STAT3C-mediated oncogenic events^47^. Similarly, a study using a S727 phosphomimetic mutant (YF/SE) resulted in STAT3 activation that enhanced anchorage-independent growth in vitro and tumorigenesis in vivo, whereas YF/SA phosphoablative mutant impaired the oncogenic capacity of STAT3 in models of prostate cancer^41^.

Similar to our results, STAT3 is also constitutively activated in macrophages in a pS727—dependent manner^34^. Another study showed that Y705 was not phosphorylated in 18 human endometrial cancer cell lines tested, while pS727 was highly present. These authors also showed that treatment with STAT3 inhibitor HO-3867 decreases pS727 levels and induces cell cycle arrest and apoptosis, suggesting that pS727—independently of Y705 phosphorylation—exerts an important role in oncogenesis in endometrial cancer^21^. Moreover, it has been shown that inhibition of PKCε by specific siRNA blocks S727 phosphorylation, STAT3 DNA binding, and gene expression as well as cell migration in prostate, skin melanoma, glioma, bladder, colon, pancreatic, and breast cancer cell lines^23^. Interestingly, results from our group demonstrated that S727 but not Y705 phosphorylation levels constitute an independent prognostic factor for overall survival in ccRCC patients^28,29^. Altogether, these studies reinforce our results by demonstrating that pS727 has an independent role from Y705 in driving tumor development by different mechanisms.

### STAT3 phosphorylation-dependent gene expression

Our study provides evidence of pS727 promoting a stronger transcriptional effect than pY705, as multiple comparisons of microarray data showed a gradual transition in gene expression patterns. The lowest number of DEGs obtained in YF/SA (Y/S) and SA (Y^P^/S) mutants (144 and 132, respectively) suggests that the effect of pY705 in our ccRCC cellular model is mild, since the only difference between these two mutants is the phosphorylation state of Y705. Moreover, the lack of significant differences between S727 phosphoablative: YF/SA (Y/S) versus SA (Y^P^/S) and phosphomimetic: YF/SD (Y/S^Pm^) versus SD (Y^P^/S^Pm^) mutants in all traits studied (except for proliferation and colony formation assay, respectively) suggest that the effects observed are independent of the Y705 phosphorylation state.

On the other hand, YF/SD (Y/S^Pm^) and SD (Y^P^/S^Pm^) mutants exhibited the highest number of DEGs (342 and 455, respectively) indicating that pS727 substantially increases STAT3 activity. Moreover, YF/SD (Y/S^Pm^) and SD (Y^P^/S^Pm^) were found associated with processes considered hallmarks of cancer such as migration, proliferation, cell adhesion, apoptosis, angiogenesis, response to stress, and wound healing among others. Remarkably, these overrepresented pathways correlated to functional experiments, where mutants carrying pS727 showed an exacerbated tumoral phenotype. Indeed, we previously showed that higher expression of pS727 levels constitutes an independent prognostic factor of overall survival of ccRCC patients and, possibly, a novel biomarker to stratify patients for adjuvant therapy^28,29^. Moreover, the present study demonstrates that STAT3 activation through S727 phosphorylation is responsible for the expression of a subset of target genes that promote a more aggressive tumoral phenotype in ccRCC. Our data is further supported by previous reports describing that phosphorylation at both sites, pY705 and pS727, produces an optimal STAT3 transcriptional activity which, additionally, results in an increased number and size of anchorage-independent colonies^27^. Concordantly, our phosphoablative mutants for Y705 (YF) and S727 (SA) both diminished the ability of cells to grow in soft agar, adding additional evidence that full activation of STAT3 is necessary to drive oncogenic transformation.

Further analyses of the biological significance of our transcriptomic results revealed that unphosphorylated STAT3 (YF/SA, Y/S) exhibits the strongest effects on processes related to IFNγ and immune defense responses. These results might indicate that in an in vivo context, where the immune system would be also present, impairment of STAT3 phosphorylation might serve to amplify the antitumor immune response and drive the clinical response to immune checkpoint blockade therapy^47,48^. These traits together with the phenotypic effects described in our cell models strongly suggest that low STAT3 phosphorylation levels must be associated with a better cancer prognosis. The mechanisms underlying the impact of unphosphorylated STAT3 on gene expression have not been addressed but deserve further studies. Contrarily, these biological processes are less represented in the YF/SD (Y/S^Pm^) and SD (Y^P^/S^Pm^) phosphomutants. Enrichment Map pathway analysis using selected S727-dependent gene datasets also showed downregulation of gene sets and pathways related to the processing of exogenous antigens and other immune effector processes, which indicates that pS727 might strongly contribute to the STAT3-related immunosuppressive effects in tumor-associated immune cells^11^. Our results suggest that targeting pS727 STAT3 may enhance anti-cancer immune responses and rescue the suppressed immunologic microenvironment in tumors. Taken together, pS727 STAT3 may represent a promising target in cancer immunotherapy.

Dysregulated metabolism is a hallmark of cancer whereby tumors reorganize their metabolism to produce sufficient energy and biosynthetic building blocks for malignant cell proliferation. Although this is not true for all cancers, ccRCC is considered a good example of the Warburg effect (defined as an increase in the rate of glucose uptake and preferential production of lactate) which is driven by a metabolic shift to aerobic glycolysis followed by lactic acid fermentation as the major form of energy production during normoxia^49^. Furthermore, a metabolic profiling study of ccRCC identified 319 differentially expressed metabolites between tumor and normal tissue samples, most of them involved in amino acid metabolism (except for cysteine, glutamate, and glutamine)^50^. Accordingly, DEGs identified in SA mutant (Y^p^/S) were mainly related to proline metabolism and transport. Alterations in proline metabolism have been associated with carcinogenesis and L-proline is emerging as a key pathway in the metabolic rewiring that sustains cell proliferation, survival, and metastatic spread in a wide variety of malignancies^51^. The extracellular matrix (ECM) represents a great reservoir of amino acids and is particularly rich in glycine and proline. This last has been recognized as relevant in the control of cancer plasticity^2^. Results presented in this study (Fig. 7B) clearly indicate the relevance of S727 STAT3 in proline metabolism since 10 of 15 overrepresented GO terms in the SA comparison were related to proline import and transport across plasma membrane and proline regulation. We also observed that transcriptional nuclear activity promoted by the pY705 residue is required to observe the impact of S727 on proline metabolism-related processes. Therefore, biological significance in SA mutant (Y^P^/S) suggested that pY705 cooperates—at least partially—to sustain malignant transformation in ccRCC.

An increase in lipid and glycogen-rich cytoplasmic deposits is a ccRCC histological feature, further supporting the concept of ccRCC as a metabolic disease. Recently, it has been shown that deficiency of chromatin modifier KDM5C in ccRCC promotes tumorigenicity by reprogramming glycogen metabolism^52^. In addition, it has been reported that the amount of adipose tissue, mainly visceral, in patients is significantly related to the presence of mutations in *VHL* and *KDM5C* genes, both drivers of ccRCC development^53^. The abnormal secretion of adipokine, proinflammatory cytokines, and growth factors in obese patients play an active role in the development of ccRCC. Leptin, mainly produced in the white adipose tissue and secreted in proportion to fat mass, has been found associated with poor clinical outcomes and ccRCC progression^54^. The fact that leptin differentially regulates S727 but not Y705 activation in ob/ob mouse adipose mesenchymal stem cells, increasing inflammatory marker MCP-1^55^, further supports the impact of STAT3 activation via pS727 on ccRCC progression.

## Conclusions

In summary, the present study constitutes the first analysis of the effects of different STAT3 phosphorylated forms in the ccRCC context. Our results provide important insights into STAT3 transcriptional behavior and indicate that pS727 activates a subset of STAT3-dependent genes that might play an important role in ccRCC development and progression.

The reported correlation found between pS727 phosphorylation and the overall survival of ccRCC patients, together with the in vitro assays presented here, supports this concept. We propose that pS727-dependent genes, acting as nodes of several oncogenic signaling pathways downstream pS727, might represent novel targets and solutions for better prognosis and treatment of ccRCC patients.

## Methods

### Cell culture

Cell lines used in this study were obtained from the ATCC® (American Type Culture Collection, Rockville USA) and were authenticated by STR analysis before experiments were carried out. Human-derived ccRCC cell lines 769-P (CRL-1933) and 786-O (CRL-1932), as well as lentiviruses producer HEK293T/17 (CRL-11268) cell line, were cultured in Dulbecco’s Modified Eagle Medium (Gibco #42430082) medium supplemented with 10% fetal bovine serum (FBS) (Gibco #10270098), 1% sodium pyruvate [100 mM] (Gibco #11360039), 1% antibiotic/antimycotic (Gibco #15240-062), and 0.2% plasmocin (Invivogen #ant-mpp) at 37 °C with 5% CO2. Culture medium was also supplemented with antibiotics puromycin (Invivogen #ant-pr-1) and blasticidin (Invivogen #ant-bl-1) to select and maintain cell lines transduced with silencing and overexpression vectors, respectively. Antibiotic concentration was determined for each cell line and cells were routinely tested for mycoplasma by PCR.

### Treatments

All experiments were carried out in the presence of 10 ng/ml IL6 (Peprotech #200-06) diluted in the corresponding culture medium unless another concentration is described in the figure legend.

### STAT3 silencing

Endogenous *STAT3* was silenced by stable lentiviral transduction of 5 different shRNA (MISSION Sigma Aldrich #TRCN0000329888, #TRCN0000329886, #TRCN0000329887, #TRCN0000020840 and #TRCN0000353630) cloned into pLKO.1-puro vector and called sh1-sh5, respectively. For lentiviruses production, HEK293T/17 cells were transiently co-transfected with the following third-generation lentiviral plasmids: VSVG (envelope), pRSV- Rev (Rev-packaging), PKPGIR (Gag/Pol-reverse-expressing), and pLKO.1-puro (transfer vector containing shRNA) in a 1:1:2:4 proportion, respectively. After 48 h of co-transfection, supernatant from HEK293T/17 cells was harvested, supplemented with 10% FBS, 1% non-essential amino acids (Biological Industries #01-340-1B), and 8 μg/ml polybrene (Sigma Aldrich #TR-1003). Supplemented supernatant was filtered through a 0.22 µm pore (Millipore #SLGS033SS) and added to ccRCC cell lines. This process was repeated at 72 h post- HEK293T/17 transfection and followed by antibiotic-mediated selection.

### STAT3 rescue and generation of phosphomutants

To reintroduce STAT3 into cell lines, a rescue mutant was created over the canonical form of human *STAT3* gene (NM_139276.3) cloned into pLX304-blast vector (Harvard PlasmID #HsCD00420180). This strategy consisted of 7 synonymous mutations (c.144C > T, c.147A > G, c.150A > G, c.153A > C, c.156 T > C, c.159C > T, and c.162 T > C) upon the sequence recognized by the selected shRNA (sh2: 5′-GCAAAGAATCACATGCCACTT-3’) to escape from the silencing mechanism previously incorporated. Over this rescue STAT3 WT form, the following STAT3 phosphomutants were introduced: phosphoablative p.Tyr705Phe (c.2114A > T) and p.Ser727Ala (c.2179 T > G), as well as phosphomimetic p.Ser727Asp (c.2179 T > G and c.2180C > A). All mutants were generated using the QuikChange Lightning Site-Directed Mutagenesis Kit (Agilent Technologies #210518) following the manufacturer’s instructions. Double mutants (p.Tyr705Phe/p.Ser727Ala and p.Tyr705Phe/p.Ser727Asp) were produced by subsequent site-directed mutagenesis reactions. All constructs were validated by sequencing and stably transduced intro previously STAT3-silenced ccRCC cell lines using lentiviral particles produced by HEK293T/17 cells in the same manner as STAT3 silencing procedure.

### RNA extraction and RT-qPCR

To isolate RNA from cells, the TRIzol reagent (Invitrogen #15596026) was used following the supplier’s instructions. Once RNA was purified, the one-step High-Capacity RNA-to-cDNA kit (Applied Biosystems #4387406) was used to retrotranscribe 2 µg RNA into complementary DNA (cDNA). For each gene tested, 20 ng of cDNA were amplified using its corresponding TaqMan probe and the TaqMan Gene Expression Master Mix (Applied Biosystems #4369016). The following TaqMan probes were used: *STAT3, KRT20, FN1, ANKS4B, ALPK2* and *TBP* (Applied Biosystems #Hs00374280_m1, #Hs00300643_m1, #Hs01549976_m1, #Hs00540939_m1, #Hs01085414_m1, and #Hs00427620_m1; respectively). Data were normalized using *TBP* gene as the internal control. Relative quantification was based on the comparison of the threshold cycle (Ct) values and expressed as the ratio of gene signal relative to the internal control signal (2^−ddCt^).

### Western Blot

Total proteins purification from cells was performed using the RIPA buffer (150 mM NaCl, 5 mM EDTA pH 8.0, 50 mM Tris–HCl pH 8.0, 1% NP-40, 0.5% sodium deoxycholate, and 0.1% SDS) supplemented with 1:200 protease inhibitor cocktail (PIC) (Sigma Aldrich #S8820), 1:200 Na3Vo4 (Sigma Aldrich #S6508), and 1:1000 NaF (Sigma Aldrich #S7920). Protein concentration was determined by the Pierce BCA protein assay (Thermo Scientific #23,225) following the manufacturer’s instructions and 20 µg of whole-cell extracts were resolved in 10% SDS-PAGE gels and transferred into 0.2 µm PVDF membranes (Millipore #ISEK00010). Membranes were blocked with 5% non-fat dry milk (NFDM) (Biorad #1,706,404) diluted in TBS-T (50 mM Tris–HCl, 150 mM NaCl, and 1% Tween-20) for 1 h. Immunodetection of the proteins of interest was performed by incubation overnight (O/N) at 4 °C with the following primary antibodies: 1:1000 STAT3 (Cell Signaling #12640), 1:1000 pY705-STAT3 (Cell Signaling #9138), 1:1000 pS727-STAT3 (Cell Signaling #9134), 1:5000 β-Tubulin (Sigma Aldrich #T4026), and 1:5000 MAPK ERK1/2 (Merck Millipore #06-182). Gels were cut prior to hybridization with antibodies during blotting, images for all replicate blots are included as Supplementary material. Membranes were then incubated for 1 h at room temperature (RT) with the corresponding secondary antibodies: 1:5000 α-Mouse IgG/HRP (Dako #P0260) and 1:5000 α-Rabbit IgG/HRP (Dako #P0448). Visualization of proteins by chemiluminescence was carried out by soaking the membrane with 1 ml Immobilon Forte HRP substrate (Merck Millipore #WBLUF0100) for 5 min at RT and exposed on the Odyssey Fc Imaging System (Li-cor). Full blots for each antibody are provided as supplementary material to confirm their specificity (Supplementary material_blots of Supplementary Fig. 2A).

### Luciferase-reporter assay

To evaluate transcriptional activity, 1.25 × 10^5^ cells per well were seeded in a 24-well plate and transfected with the Cignal dual luciferase reporter assay (Qiagen #CCS-9028) using Lipofectamine 3000 (Thermofisher #L3000008) following the suppliers’ instructions. Plasmid used included (1) an inducible reporter encoding firefly luciferase joined to tandem repeats of STAT3 transcriptional response element (TRE), (2) a negative control bearing a non-inducible reporter, and (3) a positive control constitutively expressing GFP. All plasmids were mixed with an expressing renilla luciferase construct under the control of a CMV promoter. Forty-eight hours after transfection, cells were treated with 10 ng/ml IL6 and incubated O/N at 37 °C with 5% CO2. Luciferase activity was measured using the Dual-Luciferase Reporter Assay (Promega #E1910) as recommended by the manufacturer. Luminescence of both, luciferase and renilla, was measured using the Synergy Mx luminometer (BioTek) and results were expressed as luciferase activity normalized by internal renilla (relative light units, RLU).

### Cell proliferation (XTT) assay

Cell proliferation expressed as cell viability was determined at 72 h after seeding 2.5 × 10^3^ cells per well in a 96-well plate and following the supplier’s instructions of the Cell Proliferation Kit II (Roche #11465015001). Cell growth was calculated by subtracting the absorbance of each sample at 630 nm (reference wavelength background) to the absorbance at 490 nm.

### Migration (wound-healing) assay

To evaluate cell migration, the culture-inserts 2 wells (Ibidi #81176) were used to seed 3.5 × 10^4^ cells per well divided by a gap of 500 µm in a P12-well plate. Once cells reached 100% confluence, the culture-insert was removed, and the culture medium was replaced with fresh one without (W/O) FBS to inhibit proliferation and promote only migration. Cells were then incubated for 24 h in the Cell R live cell microscope (Olympus #TIRFM) and photographs were taken at 0 and 24 h. Cell migration was calculated as the percentage of the total wound area (area to close) using ImageJ software^56^ and values were plotted at each time point.

### Clonogenic assay

To evaluate clonogenic capacity, a very low density of cells per well (1.0 × 10^3^) were seeded in a P6-well plate and incubated for 7 days. After incubation, cells were fixed with 500 μl of 4% paraformaldehyde (PFA) (Electron Microscopy Science #15714) and stained with 500 μl of 0.1% crystal violet (Sigma Aldrich #V5265) for 15 min at RT, respectively. Finally, cells were thoroughly washed and lysed with 500 μl of 1% SDS (Panreac #A0675) by gentle shaking at 100 rpm for 15 min at RT. Clonogenicity was measured as absorbance at 590 nm.

### Anchorage-independent growth (soft-agar) assay

First, to avoid cell attachment from the plate, P24-well plates were coated with 500 μl of 0.6% Noble Agar (Affymetrix #J10907) and incubated for 30 min at RT. Then, 2.0 × 10^4^ cells per well diluted in 500 μl of 0.3% Noble Agar were seeded onto the bottom layer and incubated for 30 min at RT to solidify. Cells were left to grow for 28 days and fed with 100 μl of culture medium every three days. At the end of the experiment, cells were stained with 100 μl of 1 mg/ml nitro blue tetrazolium chloride (Abcam #AB146262) O/N at 37 °C, and photographs were taken to count colonies with the software OpenCFU (Quentin Geissmann).

### Microarray

Cells were treated with 10 ng/ml IL6 (Peprotech #200-06) for 48 h and then RNA was isolated as indicated before. RNA quality was evaluated in the BioAnalyzer 2100 (Agilent Technologies #G2939BA) in a nano-chip format. Briefly, 200 ng of total RNA were retrotranscribed into cDNA using the WT pico HT kit (Thermo Fisher #902622). The resulting cDNA was fragmented, labeled, and hybridized using the GeneAtlas Hybridization, Wash, and Stain Kit for WT Array Strips (Thermo Fisher #901667), and finally, hybridization was performed using the Clariom S array (Applied Biosystems #902926) and scanned in the GeneTitan MC System (Affymetrix #00-0373). The analysis was based on five biological replicates per experimental condition. To make results comparable, as well as to remove technical biases, microarray raw data underwent quality control to identify potential outliers and was corrected for batch effects. To increase statistical power and reduce unnecessary noise, genes with low standard deviations (below 65 percentile) were removed as not to be considered differentially expressed. To deal with multiple testing issues, *p* values were adjusted to control the False Discovery Rate (FDR) using the Benjamini and Hochberg method^57^.

### Biological significance analysis

Analysis of biological significance was performed in the webserver g:Profiler (Ensembl 93, Ensembl Genomes 40 (rev 1760, database built on 2018-10-02)) using filtered (adj.P.Value  < 0.25, logFC > 0.5) and ranked lists (according to adj.P.Value) of differentially expressed genes from microarray analysis. Overrepresented biological functions (BF) were determined by using the Gene Ontology (GO) database and electronic GO annotations were discarded. It was considered statistically significant when adj.P.Value < 0.05. The protocol followed is thoroughly described in^58^.

### Gene set enrichment analysis (GSEA) based pathway enrichment analysis

Pathway enrichment analysis was carried out by searching for enriched gene sets using GSEA software, as previously described^58^. The pathway gene set definition (GMT) files loaded on the software were created with the archived instance of g:Profiler previously described. “Gene set permutation” with 1000 permutations to compute *p* Values was used for enriched gene sets followed by GSEA’s standard multiple testing correction.

### Enrichment map pathway analysis visualization

Enrichment results were visualized using the Enrichment Map plugin for the Cytoscape network visualization and analysis software (3.9.1 version). The following apps were used: EnrichmentMap, clusterMaker, WordCloud, NetworkAnalyzer, and AutoAnnotate^58^. Enrichment maps were constructed by uploading GSEA individual dataset using an FDR threshold of 0.1. Enrichment maps symbolize each gene set as a node in the network. Pathways are shown as circles (nodes) and, if the pathways share many genes, they are connected with lines (edges). Nodes are colored by enrichment score (ES) where blue and red meaning down- and upregulated pathways, respectively. Edges are sized based on the number of genes shared by the connected pathways. Network layout and clustering algorithms automatically group similar pathways into major biological themes.

### Statistical analysis

All data sets were first tested for normality (Kolmogorov–Smirnov test) and statistical significance was determined by Student’s t-test or one-way analysis of variance (ANOVA) followed by Turkey’s post hoc test. For data not following normal distribution, the Mann–Whitney’s unpaired t-test or the non-parametric ANOVA followed by Dunn’s post hoc test were used. All statistical analyses were performed using GraphPad Prism 6 software (RRID: SCR_002798). Values are expressed as mean ± SD and results were considered statistically significant when *p < 0.05, **p < 0.01, or ***p < 0.001.

### Ethical approval

No human data or samples were used in this study. All experimental procedures were carried out in accordance with European research regulations (PE/12/2021/INIT). As established by current European regulation (PE/53/2018/REV/1 and PE 17 2016 INIT), generated data will be stored for a minimum of 15 years.

### Supplementary Information

Supplementary Information.

## Data Availability

The datasets used and/or analyzed during the current study will be deposited in NCBI’s Gene Expression Omnibus upon acceptance and will be accessible through GEO Series accession number GSE245862 (http://www.ncbi.nlm.nih.gov/geo/query/acc.cgi?acc=GSE245862).
