# Supplementary material for: STAT3 phosphorylation at serine 727 activates specific genetic programs and promotes clear cell renal cell carcinoma (ccRCC) aggressiveness
Source: Sci Rep. 2023 Nov 9;13:19552. doi: 10.1038/s41598-023-46628-5 (PMC10636117; doi:10.1038/s41598-023-46628-5)

SUPPLEMENTAL MATERIAL

STAT3 phosphorylation at serine 727 activates specific genetic programs and promotes clear cell renal cell carcinoma (ccRCC) aggressiveness.

Arévalo J[1,*], Campoy I[1], Durán M[1], Nemours S[2], Areny A[1], Vall-Palomar M[1], Martínez C[3], Cantero-Recasens G[1], and Meseguer A[1,4,*].

1. Renal Physiopathology Group, Vall d'Hebron Research Institute, Passeig Vall d'Hebron 119-129, 08035 Barcelona, Spain.
2. Molecular Oncology Group. Biodonostia Health Research Institute. Paseo Dr. Begiristain, s/n, 20014 San Sebastián, Gipuzkoa.
3. Vascular and Renal Translational Research Group. Lleida Institute for Biomedical Research Dr. Pifarré Foundation (IRBLleida). Av. Alcalde Rovira Roure, 80, 25198, Lleida, Spain.
4. Departament de Bioquímica i Biologia Molecular, Unitat de Bioquímica de Medicina, Universitat Autònoma de Barcelona, Bellaterra, Spain.

* Correspondence to:

Jazmine Arévalo: jazmine.arevalo@vhir.org (+34 932746200)

Anna Meseguer: ana.meseguer@vhir.org (+34 934894061)

INDEX

Supplementary figures

Supplementary figure 1 3
Supplementary figure 2 4
Supplementary figure 3 5
Supplementary figure 4 6

Supplementary tables

Supplementary table 1 7
Supplementary table 2 7
Supplementary table 3 7
Supplementary table 4 9

Supplementary material

Uncropped gels 13

SUPPLEMENTARY FIGURE 1

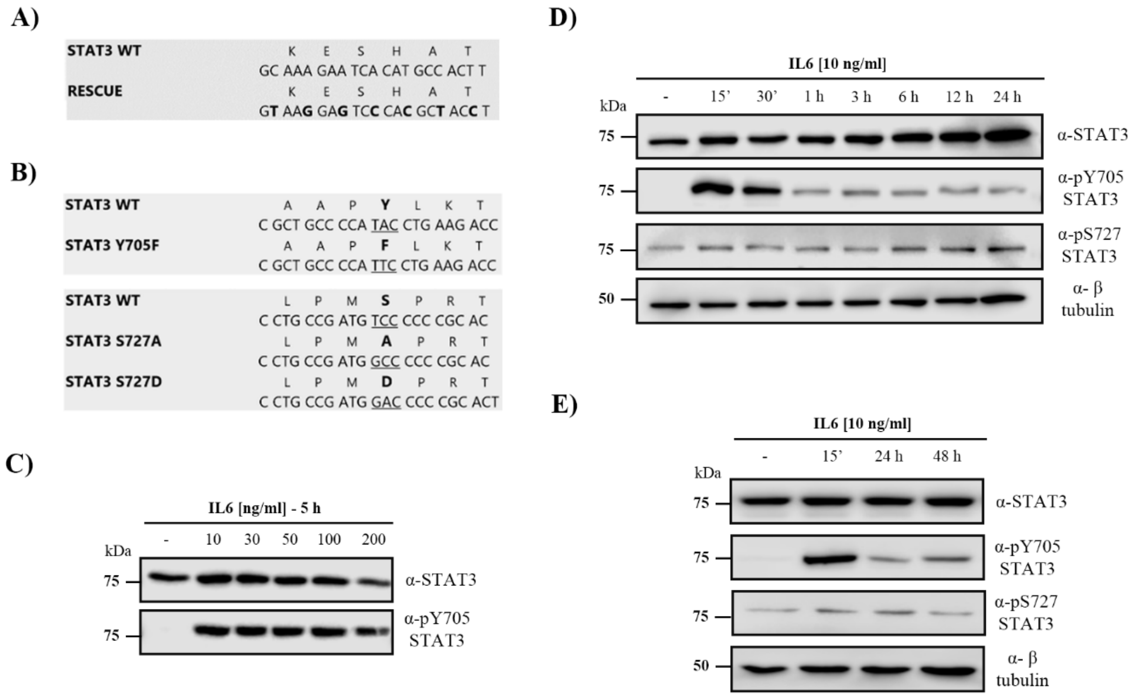

Supplementary figure 1. Generation of the cellular models and effect of IL6 on STAT3 in the 769-P cell line. STAT3 sequence with the mutations introduced to generate **A)** the rescue (to avoid RISC-mediated degradation of the mRNA) and **B)** the different phosphomutants. **C)** Representative western blots (WB) of IL6 stimulation at different concentrations for a period of 5 hours (0, 10, 30, 50, 100, and 200 ng/ml). **D)** and **E)** Representative WB of 10 ng/ml IL6 stimulation at different times (15', 30', 1 h, 3 h, 6 h, 12 h, 24 h, and 48 h) in 769-P cells.

SUPPLEMENTARY FIGURE 2

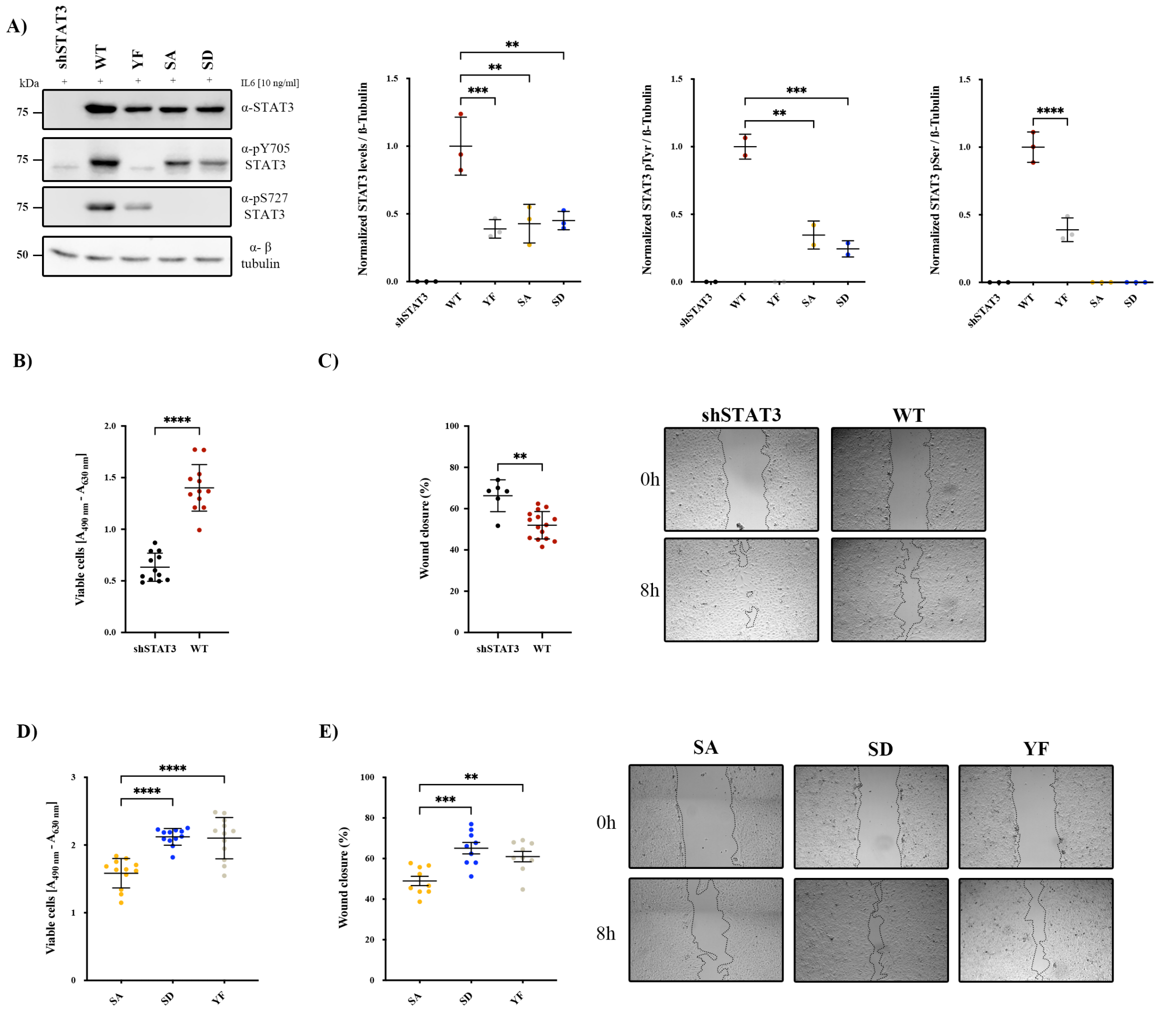

Supplementary figure 2. Generation of cellular models of STAT 3 phosphomutants in 786 O cells and their tumoral phenotype. **A)** Representative western blot showing protein levels of reintroduced STAT3 forms detected by specific antibodies against total STAT3 and the phosphorylated forms of Y705 and S727 residues. Treatment with 10 ng/ml IL 6 for 30 min was used as classical activator of the canonical STAT3 pathway through Y705 phosphorylation. Densitometric analysis of three independent western blots showing relative levels of total STAT3, pY705, and pS727 normalized against β-tubulin (loading control). Data was compared to the wild type form (WT). **B)** and **D)** Proliferation capacity was evaluated at 72 h by subtracting the absorbance of each sample at 630 nm (reference wavelength background) from the absorbance at 490 nm. **C)** and **E)** Cell migration was evaluated by wound healing assay. Wound closure was registered, and pictures were taken at 0 and 24 h. The percentage of the total wound area closed was calculated using ImageJ software. All experiments were carried out in the presence of 10 ng/ml IL6. Dots represent individual values and error bars indicate the mean ± SD, n= 3. Statistical significance *p<0.05, **p<0.01, and ***p<0.001.

SUPPLEMENTARY FIGURE 3

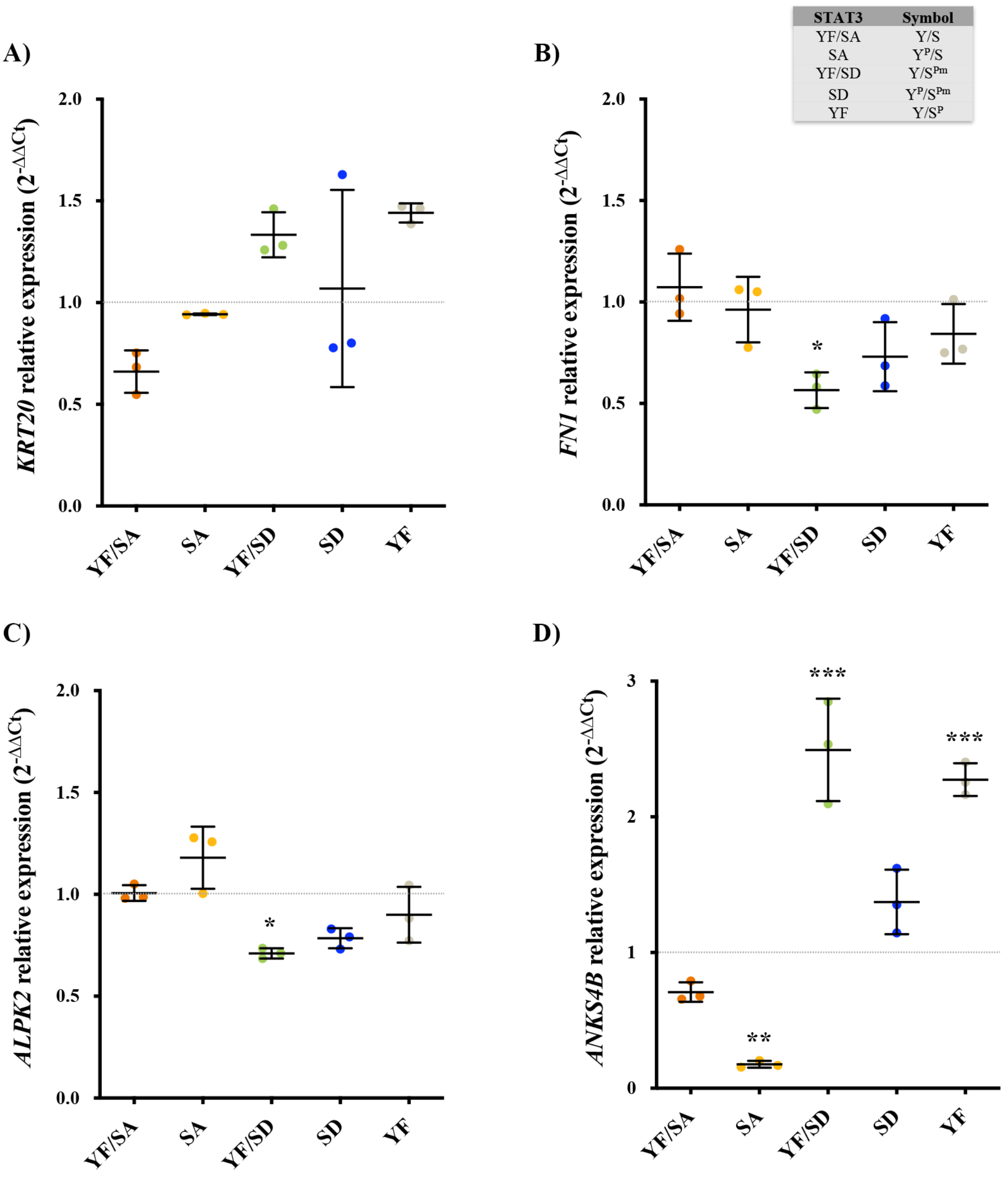

Supplementary figure 3. Validation of microarray results in the ccRCC cell lines 769-P by RT-qPCR. Relative expression levels of **A)** *KRT20*, **B)** *FN1*, **C)** *WNT7A*, and **D)** *ANKS4B* genes assessed by RT-qPCR. Data were normalized using TBP gene as the internal control and quantification relative to shSTAT3 was performed by the $2^{-\Delta\Delta Ct}$ method. The expression pattern of the selected genes agrees with differential gene expression found in microarrays (see Supplementary table 2). The dotted line (y=1) represents shSTAT3 normalized values. Dots represent individual values and error bars indicate the mean ± SD, n=3. Statistical significance of comparisons vs. shSTAT3, * $p<0.05$, ** $p<0.01$, and *** $p<0.001$.

SUPPLEMENTARY FIGURE 4

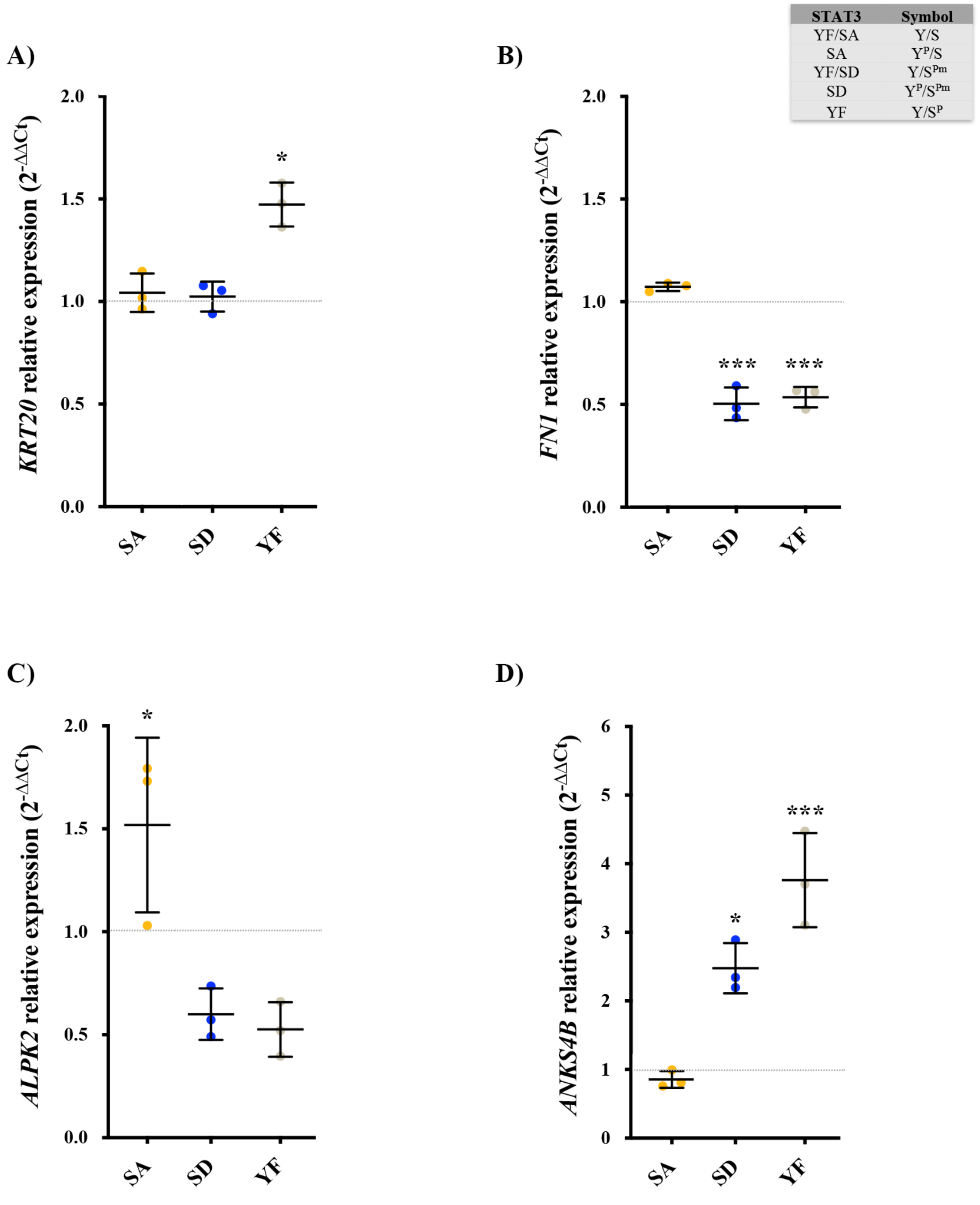

Supplementary figure 4. Validation of microarray results in the ccRCC cell lines 786-O by RT-qPCR. Relative expression levels of **A)** *KRT20*, **B)** *FN1*, **C)** *WNT7A*, and **D)** *ANKS4B* genes assessed by RT-qPCR. Data were normalized using TBP gene as the internal control and quantification relative to shSTAT3 was performed by the $2^{-\Delta\Delta Ct}$ method. The expression pattern of the selected genes agrees with differential gene expression found in microarrays (see Supplementary table 2). The dotted line (y=1) represents shSTAT3 normalized values. Dots represent individual values and error bars indicate the mean ± SD, n=3. Statistical significance of comparisons vs. shSTAT3, * p<0.05, ** p<0.01, and *** p<0.001.

SUPPLEMENTARY TABLES

Supplementary table 1. STAT3 logFC values from the microarray in 769-P cells.

Comparison	STAT3 expression	
	logFC	FDR
YF/SA vs shSTAT3	4.93790	2.09E-39
SA vs shSTAT3	5.05620	7.12E-40
YF/SD vs shSTAT3	5.09523	5.02E-40
SD vs shSTAT3	5.14054	3.35E-40
YF vs shSTAT3	5.07374	6.08E-40

Supplementary table 2. Expression values from the microarray in 769-P cells.

Gene Symbol	YF/SA vs shSTAT3		SA vs shSTAT3		YF/SD vs shSTAT3		SD vs shSTAT3		YF vs shSTAT3	
	logFC	*FDR*	*logFC*	*FDR*	*logFC*	*FDR*	*logFC*	*FDR*	*logFC*	*FDR*
KRT20	-0.5778	1.20E-01	0.5083	9.03E-01	0.8143	1.95E-04	0.5337	7.73E-01	0.8599	9.61E-02
FN1	-0.5067	7.79E-01	-0.5595	6.12E-01	-1.3356	1.89E-09	-0.8035	1.08E-04	-1.5816	1.75E-11
ALPK2	0.5225	9.34E-01	0.6766	1.86E-02	-1.2216	7.18E-07	-1.0268	2.30E-05	-1.6964	1.29E-02
ANKS4B	0.5608	8.73E-01	-1.1794	2.17E-03	1.4402	2.25E-05	1.1861	4.17E-04	1.0718	1.46E-01

Supplementary table 3. DEGs regulated by S727 phosphorylation in 769-P cells.

Upregulated genes			Downregulated genes		
Gene Symbol	logFC	FDR	Gene Symbol	logFC	FDR
NR0B2	1.2278	1.97E-07	IFITM3	-1.0549	2.08E-07
TFEC	1.0117	8.49E-03	SULT1C2	-0.9452	1.60E-03
LGALS2	0.9299	3.94E-04	MSC	-0.9148	2.80E-04
PADI3	0.8817	1.38E-02	MCOLN3	-0.8748	2.70E-03
HHLA2	0.8443	3.18E-02	PREX2	-0.8628	1.65E-05
F2RL1	0.8263	9.30E-05	MOK	-0.8624	7.33E-05
KRT20	0.8143	1.95E-04	IFITM1	-0.8370	4.35E-04
PRSS8	0.7803	1.57E-02	ICAM1	-0.8325	5.11E-04
LRRC19	0.7667	6.86E-02	IFITM2	-0.8052	8.29E-05
SLC3A1	0.7383	2.86E-04	NNMT	-0.7912	1.90E-03
TOX3	0.7304	4.53E-03	MBNL3	-0.7370	3.25E-03
VANGL2	0.7041	2.55E-03	ANTXR1	-0.7349	9.94E-05
BAIAP2L2	0.7027	5.58E-02	SLC9A7	-0.7130	1.21E-03
ZNF385D	0.7005	8.51E-02	TBC1D2	-0.6776	1.73E-03
ARSF	0.7005	1.68E-02	TTC29	-0.6748	7.57E-02
LRP2	0.6766	1.30E-01	SGK1	-0.6664	1.34E-02
TGFBR3	0.6706	5.06E-02	P3H2	-0.6564	2.97E-02
METTL27	0.6639	1.05E-02	SLC44A2	-0.6398	3.28E-03
SCNN1D	0.6551	6.52E-02	CITED2	-0.6395	1.93E-04
HTR1D	0.6431	1.38E-03	SLC16A9	-0.6259	9.51E-03

ABCC8	0.6299	3.80E-02	EPHB1	-0.6238	6.83E-02
CUBN	0.6269	1.71E-01	RRAD	-0.6180	2.48E-02
CDH20	0.6258	1.06E-01	COL4A1	-0.6158	2.65E-03
HNF4A	0.6013	1.01E-01	PTPRO	-0.6127	9.21E-02
STEAP2	0.5977	5.76E-02	TGFBI	-0.5981	4.90E-04
NEBL	0.5948	3.51E-02	WNT2B	-0.5947	4.17E-02
WAS	0.5943	7.95E-02	ADAM12	-0.5945	1.27E-02
SERPINB13	0.5941	3.00E-02	SAA1	-0.5925	9.64E-02
SLC6A17	0.5895	2.02E-02	CBR1	-0.5915	1.55E-03
TNFSF11	0.5892	7.78E-02	IFIT3	-0.5907	7.84E-04
RDH10	0.5861	5.84E-03	SULF2	-0.5857	3.25E-03
TSTD1	0.5827	1.86E-02	LYPD1	-0.5847	2.05E-03
LGALS4	0.5821	2.64E-02	GPR85	-0.5842	8.84E-02
FBXO27	0.5805	8.10E-02	B3GALT1	-0.5811	1.93E-02
KCNK5	0.5801	1.15E-01	VSTM2L	-0.5769	1.14E-01
ADH6	0.5779	7.78E-02	CLUL1	-0.5766	8.55E-02
SLC17A1	0.5737	4.51E-03	SOX14	-0.5756	4.07E-02
ABHD1	0.5682	6.48E-02	ABCA5	-0.5727	9.45E-02
FGR	0.5671	5.54E-02	SDC2	-0.5724	7.54E-04
CLIP4	0.5659	9.64E-02	ABCA1	-0.5701	3.86E-03
STOX1	0.5651	5.44E-02	CEACAM1	-0.5662	1.58E-01
ZBTB18	0.5640	2.02E-02	MRC1	-0.5573	5.16E-02
HLA-DPB1	0.5626	1.29E-01	GPC6	-0.5559	2.32E-02
DNASE2	0.5495	9.63E-03	CDKL5	-0.5540	1.79E-01
CTDSPL	0.5485	3.59E-03	CD33	-0.5536	7.38E-02
SMCR5	0.5456	6.86E-02	ENDOU	-0.5526	4.55E-02
MAL	0.5433	8.43E-02	MUC19	-0.5487	6.68E-02
PDE9A	0.5385	3.39E-02	MSRB3	-0.5468	2.32E-02
FOXA1	0.5381	2.25E-02	IRS1	-0.5448	4.77E-02
STK32A	0.5369	1.83E-01	NGF	-0.5424	6.50E-02
CREB5	0.5356	2.75E-03	KCNH1	-0.5377	1.42E-02
SLC7A2	0.5336	6.03E-02	TNFSF10	-0.5301	3.32E-03
OVGP1	0.5324	1.51E-01	IGFBP5	-0.5294	1.27E-01
ZNF683	0.5298	1.30E-01	RELB	-0.5262	1.38E-01
SLC9B2	0.5297	5.76E-02	HPGD	-0.5261	6.81E-02
PLCH1	0.5293	3.35E-02	ADH1C	-0.5242	7.38E-02
ACMSD	0.5258	8.43E-02	TMEM45A	-0.5230	9.21E-02
NEB	0.5234	1.53E-01	MYBL1	-0.5134	1.27E-02
TM4SF4	0.5196	1.11E-01	SDC4	-0.5122	5.24E-03
ZC3H6	0.5186	1.29E-01	THBS1	-0.5093	9.07E-03
SYNC	0.5160	1.30E-01	SERPINA1	-0.5077	4.76E-02
CRYBG1	0.5159	4.13E-02	MCC	-0.5067	1.26E-01
CASC10	0.5130	3.19E-02	JUNB	-0.5046	4.60E-02
PPL	0.5064	1.22E-01	ODAPH	-0.5027	1.54E-01
GPNMB	0.5062	2.08E-01	OR52B6	-0.5014	1.20E-01
PROZ	0.5015	1.56E-01	C6orf58	-0.5008	6.36E-02

Supplementary table 4. DEGs regulated by simultaneous Y705 and S727 phosphorylation in 769-P cells.

Upregulated genes			Downregulated genes		
**Gene Symbol**	**logFC**	**FDR**	**Gene Symbol**	**logFC**	**FDR**
C1QTNF3-AMACR	1.2482	3.06E-06	CSF2	-0.9179	1.39E-02
AMACR	1.2057	1.42E-05	TRAF1	-0.8922	8.25E-03
KCNJ13	1.1894	1.49E-03	CD274	-0.8847	3.94E-04
CFI	1.0827	6.11E-04	EPB41L3	-0.8821	1.37E-03
C1QL1	1.0118	3.06E-06	DOCK2	-0.8426	1.41E-03
RASSF6	0.9740	3.33E-05	RNF144B	-0.8242	1.13E-04
TCN2	0.9070	1.47E-03	TINAGL1	-0.8104	2.18E-05
SLC7A7	0.8933	1.08E-03	KRTAP2-1	-0.7939	2.58E-04
MAN1A1	0.8458	7.55E-04	IL1RAPL1	-0.7935	2.50E-02
IL1R1	0.8395	8.82E-03	VSTM1	-0.7908	2.01E-02
SLC29A3	0.8370	9.71E-04	GPCPD1	-0.7815	7.93E-03
SATB1	0.8343	3.22E-03	NUAK2	-0.7714	8.11E-05
NDRG1	0.8169	6.82E-05	ENC1	-0.7704	6.11E-04
EGLN3	0.8147	1.36E-03	NRCAM	-0.7699	2.05E-05
PRRG4	0.8016	2.51E-04	IRAK2	-0.7205	1.50E-02
SOX4	0.7967	3.38E-03	NEXN	-0.7205	9.94E-03
KCNK2	0.7951	7.22E-05	PLEKHH2	-0.6977	1.00E-02
ANGPTL4	0.7755	1.17E-02	KRTAP2-2	-0.6842	2.94E-03
ITPR1	0.7717	3.36E-03	TNFAIP8	-0.6748	3.56E-03
GJB1	0.7669	3.39E-02	LPCAT2	-0.6666	4.75E-03
ALOX5	0.7649	1.18E-03	SLC9A2	-0.6645	1.32E-02
MTMR11	0.7648	1.83E-03	ADAMTSL3	-0.6604	1.17E-02
ADAP2	0.7616	4.40E-05	EHD4	-0.6602	4.01E-03
CDKL5	0.7372	3.07E-02	NIPAL1	-0.6446	3.42E-02
GPRIN3	0.7145	6.15E-02	IDI2	-0.6394	2.70E-02
TGIF2	0.7080	1.41E-03	FBXO15	-0.6366	1.17E-02
NRP1	0.7072	4.81E-05	MYH15	-0.6112	4.03E-02
BMP4	0.6991	4.12E-02	NFE2L3	-0.6066	7.08E-03
TMEM37	0.6915	9.98E-03	IFI44	-0.6062	3.88E-02
C1QTNF6	0.6759	8.81E-04	ANK3	-0.6019	9.52E-02
SLC6A15	0.6689	1.17E-02	AMPD3	-0.5998	6.37E-02
RIPK4	0.6680	4.03E-03	NEDD1	-0.5978	1.21E-01
ADAMTS15	0.6678	6.20E-03	NKX3-1	-0.5970	2.86E-02
ZNF549	0.6675	1.26E-02	ANKRD33B	-0.5960	4.66E-03
MAP3K12	0.6637	3.42E-02	GBP7	-0.5896	2.95E-02
FAT4	0.6624	1.20E-02	ZNF703	-0.5887	1.76E-02
ZNF705D	0.6604	8.33E-02	DCLK1	-0.5881	4.62E-04
NR1H4	0.6591	3.09E-02	SSX8P	-0.5871	6.31E-02
VWA5A	0.6572	4.16E-02	GALNT10	-0.5865	1.30E-03
CA12	0.6570	1.49E-03	ANKRD45	-0.5847	8.24E-02
PRR19	0.6541	6.46E-02	MICAL2	-0.5838	4.65E-03

RHOBTB1	0.6530	3.39E-02	CD83	-0.5795	5.66E-02
EDIL3	0.6528	4.99E-02	ARID3B	-0.5786	8.17E-03
SLC39A8	0.6431	2.02E-03	N4BP3	-0.5772	5.13E-02
GUCY1B1	0.6352	2.24E-03	MAMLD1	-0.5758	8.10E-03
AK7	0.6332	1.26E-02	EPHB2	-0.5754	5.36E-03
PTPRS	0.6282	1.37E-03	CXCL8	-0.5724	2.29E-01
AASS	0.6251	8.51E-02	SEMA7A	-0.5719	9.76E-02
DYRK1B	0.6247	3.12E-03	SEPT3	-0.5581	4.13E-02
PALM2-AKAP2	0.6227	2.50E-02	GSDMC	-0.5581	7.88E-02
ASS1	0.6109	2.72E-04	FRMPD3	-0.5574	1.52E-02
STMN3	0.6090	3.78E-03	L1CAM	-0.5532	5.97E-02
CNTN4	0.6064	1.02E-01	RBMS3	-0.5491	7.54E-02
PPP1R1A	0.6051	4.07E-03	CYR61	-0.5462	1.17E-02
TSPAN33	0.6041	1.22E-02	C8orf74	-0.5458	3.37E-02
ZIM3	0.6001	1.07E-01	GJB4	-0.5420	5.68E-02
PITX2	0.5975	1.40E-02	AFAP1	-0.5418	3.30E-02
THBS3	0.5956	2.78E-02	PHGDH	-0.5408	1.32E-01
SYNE2	0.5948	1.15E-02	TMEM272	-0.5380	6.23E-02
CDKL1	0.5924	3.35E-02	INKA2	-0.5378	1.01E-01
PTH2R	0.5913	4.26E-02	C6orf99	-0.5356	3.45E-02
PKHD1	0.5898	1.73E-01	INSL3	-0.5350	5.89E-02
ARHGAP24	0.5893	5.24E-02	STARD13	-0.5348	8.43E-03
LRRC75B	0.5887	2.40E-02	P4HA2	-0.5337	4.42E-03
BNC2	0.5875	7.93E-03	IRF8	-0.5319	1.65E-01
C5	0.5857	3.35E-02	TMOD2	-0.5318	6.30E-02
NEFM	0.5839	6.57E-02	GFRA4	-0.5307	1.05E-01
SLC29A4	0.5835	2.99E-02	MB21D2	-0.5297	2.63E-02
DOC2A	0.5834	6.67E-02	ZNF432	-0.5290	9.01E-03
PACRG	0.5779	1.07E-01	CUL9	-0.5268	1.90E-01
IL1R2	0.5768	3.99E-02	ATP6V1FNB	-0.5267	2.30E-01
YPEL2	0.5750	2.24E-01	CDCP1	-0.5244	7.00E-03
C1QL3	0.5746	3.39E-02	DNAJC15	-0.5213	1.61E-03
ZNF385A	0.5725	3.42E-02	CD207	-0.5205	9.92E-02
EFCAB5	0.5720	7.18E-02	RFTN1	-0.5201	9.41E-02
KANK1	0.5705	5.10E-04	NUAK1	-0.5196	5.68E-02
FGFR3	0.5662	6.11E-02	CITED4	-0.5157	3.24E-02
PRR23A	0.5639	1.32E-02	RSPO2	-0.5149	6.11E-02
PLEKHA7	0.5624	2.28E-02	ALDH1L1	-0.5146	1.96E-01
GUCY1A1	0.5600	5.37E-03	PDGFB	-0.5136	1.66E-02
ADGRF1	0.5593	5.82E-02	BATF2	-0.5126	6.44E-02
EML5	0.5577	1.03E-01	ETS2	-0.5102	7.12E-03
GAREM1	0.5566	6.32E-03	CLEC12B	-0.5099	6.23E-02
RAB26	0.5514	6.31E-02	WDR86-AS1	-0.5048	1.33E-01
CACNB3	0.5509	3.75E-03	TLL1	-0.5048	2.25E-01
RTN4RL2	0.5505	1.57E-02	PER2	-0.5047	5.73E-02
HES1	0.5484	1.29E-02	CDA	-0.5017	4.85E-02

TLE3	0.5468	7.26E-03	GLT1D1	-0.5006	6.55E-02
HGD	0.5456	5.27E-03			
BTBD19	0.5451	1.25E-01			
WDR72	0.5415	1.50E-02			
PLD5	0.5412	8.26E-02			
UGT8	0.5404	7.84E-03			
BTG2	0.5389	1.24E-02			
OLFML2B	0.5385	1.22E-01			
CALCRL	0.5384	1.23E-01			
ATP11C	0.5374	5.33E-03			
VAMP8	0.5363	1.00E-02			
RGS14	0.5338	1.88E-02			
CATSPERG	0.5310	1.54E-01			
MYO5C	0.5294	3.12E-02			
SLC23A1	0.5270	8.36E-02			
SPAG4	0.5268	6.59E-02			
FBXO17	0.5254	6.05E-03			
RASGEF1C	0.5250	1.01E-01			
CERCAM	0.5248	1.29E-02			
DLC1	0.5246	4.56E-02			
NPY1R	0.5233	9.75E-03			
P3H2	0.5223	9.75E-02			
STK31	0.5211	9.07E-02			
ORAI3	0.5209	2.41E-02			
COX7B2	0.5205	4.42E-02			
DNMT3A	0.5202	1.33E-01			
ACY3	0.5201	1.90E-01			
CALCOCO1	0.5199	1.50E-02			
DPYSL5	0.5199	1.21E-01			
SLC12A9	0.5186	4.91E-02			
ZNF395	0.5169	7.68E-02			
APOBR	0.5167	1.01E-01			
DBP	0.5160	6.91E-02			
FAM214A	0.5152	1.62E-01			
HSD11B2	0.5149	4.90E-02			
FAM13A	0.5148	3.42E-02			
GNG8	0.5144	1.91E-01			
GNLY	0.5142	1.28E-01			
PMM1	0.5124	2.75E-02			
ST8SIA4	0.5094	1.21E-01			
ACVR2B	0.5089	3.00E-02			
C1QL4	0.5078	8.39E-02			
ARHGAP42	0.5077	8.26E-02			
SGCZ	0.5066	9.41E-02			
ZKSCAN3	0.5050	9.97E-02			

GALM	0.5038	1.76E-01	
C1orf229	0.5034	1.75E-01	
HNF1A	0.5029	3.88E-02	
IER5L	0.5027	1.27E-01	
CNKSR3	0.5022	4.16E-02	
MESP2	0.5020	5.66E-02	
LONRF1	0.5000	1.97E-02	

SUPPLEMENTARY MATERIAL

Uncropped gels for all the main and supplementary figures follow. Individual blots were run for α-STAT3 (total), α-pY705, and α-S727 since all exhibit the same molecular weight. For those gels that were cut prior to hybridization with antibodies during blotting, we provide images for all replicate blots. In addition, we include full blots for each antibody to confirm their specificity (blots of Supplementary Figure 2A). Biological replicates are indicated below each image. Blue arrows indicate where membranes were cut prior hybridization. Images surrounded by dotted lines indicate they are the same blot with different exposure times.

1. **Figure 1A**

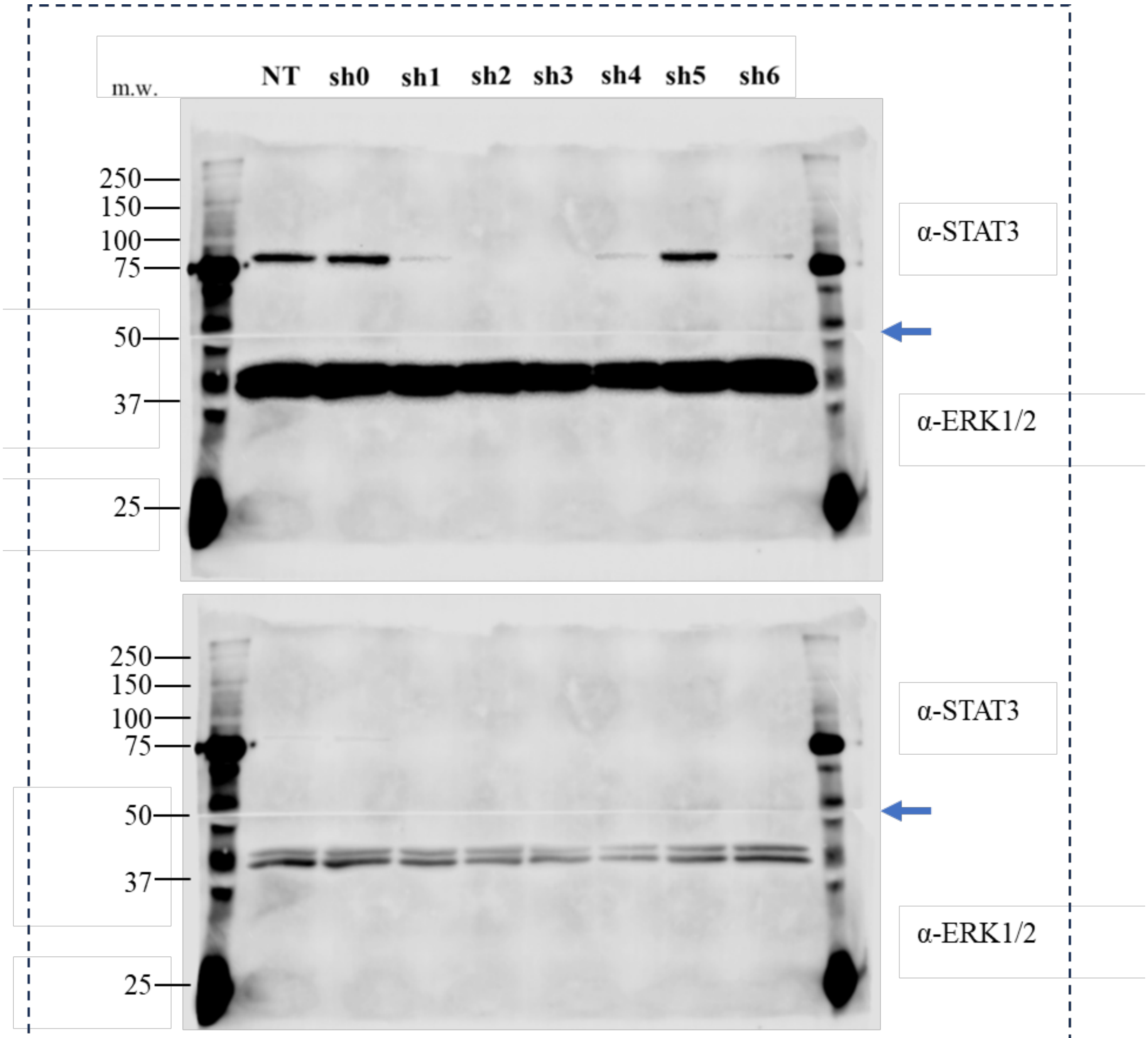

Replicate 1

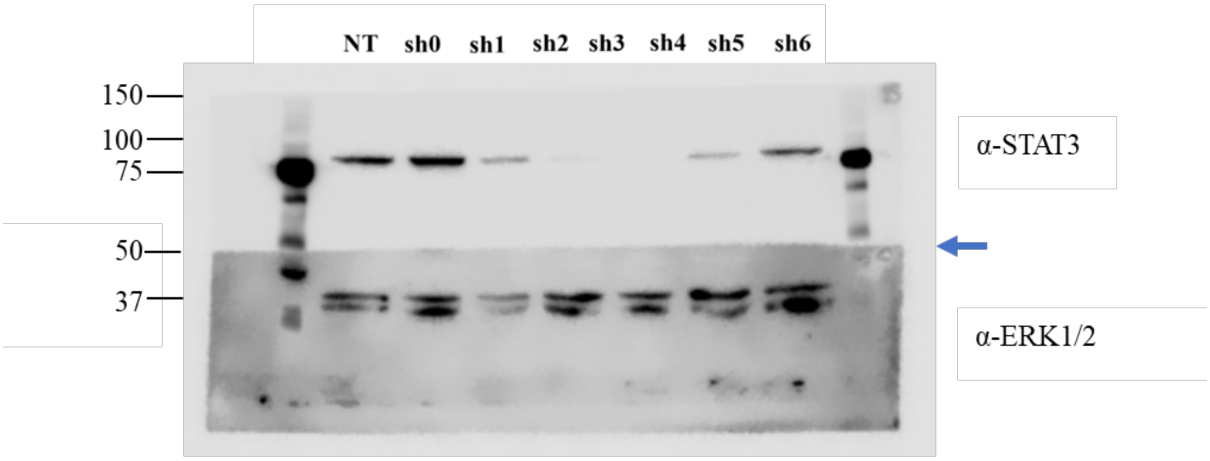

Replicate 2

2. Figure 1C

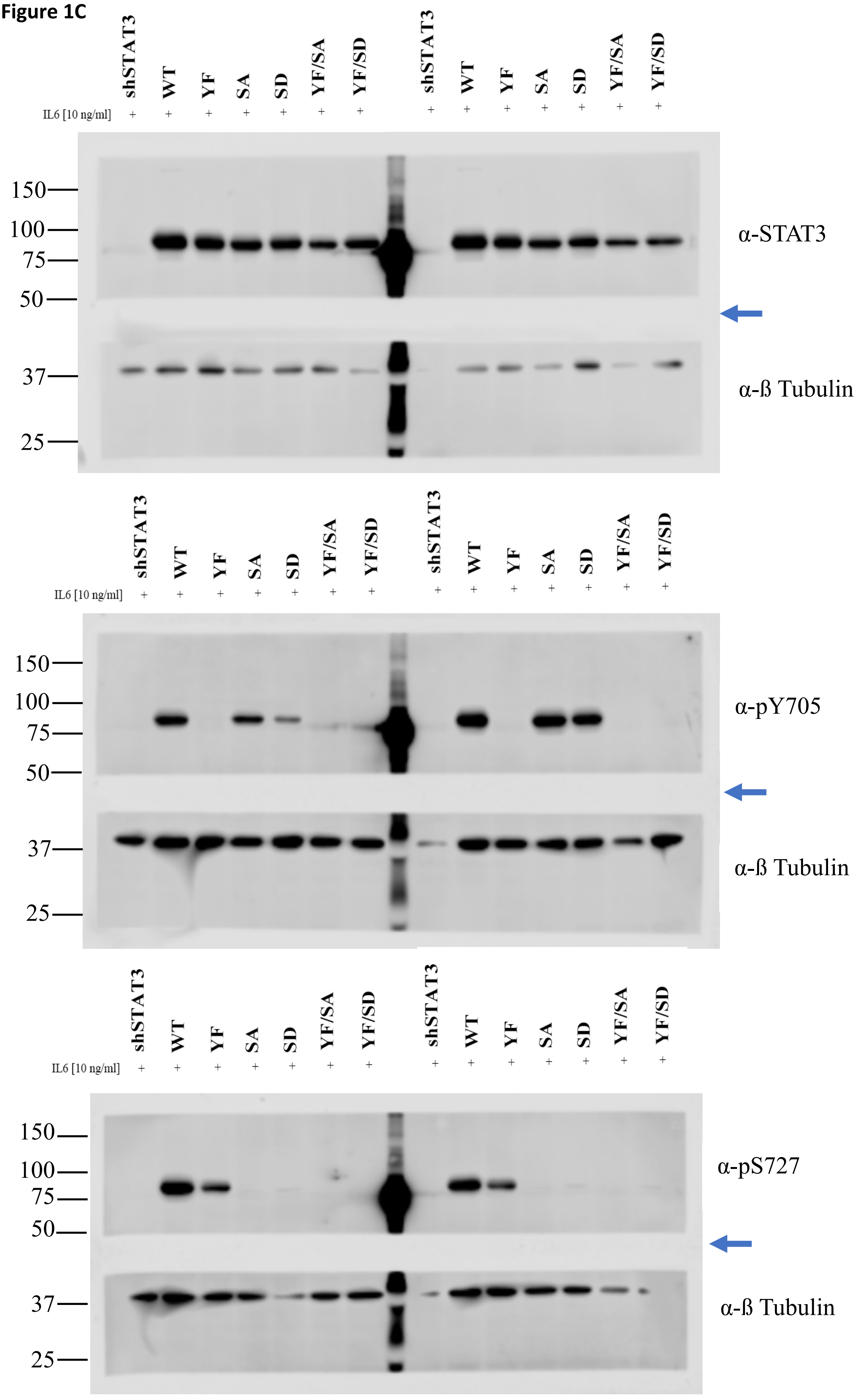

Replicate 1 Replicate 2

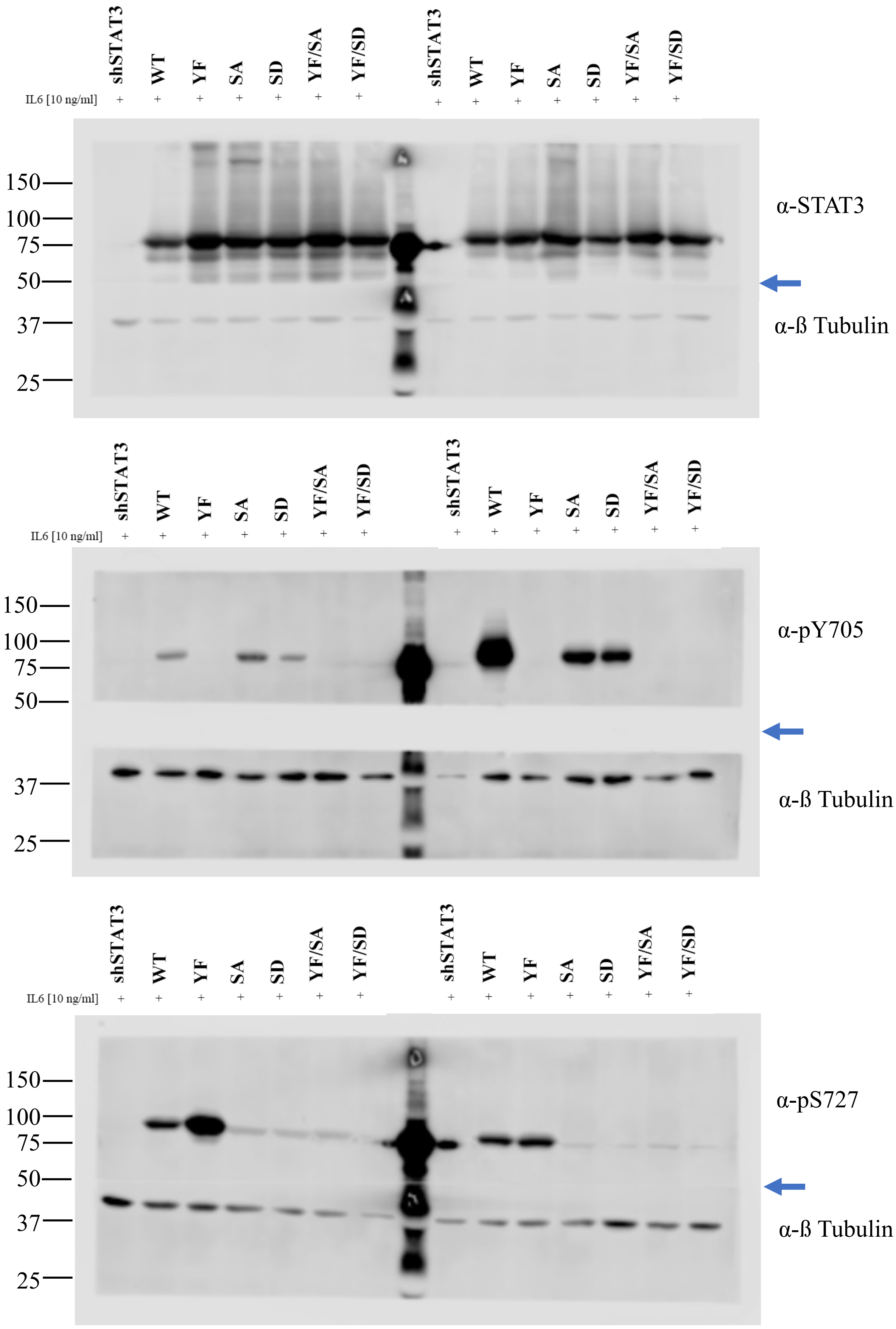

Replicate 3 Replicate 4

3. **Supplementary Figure 1C**

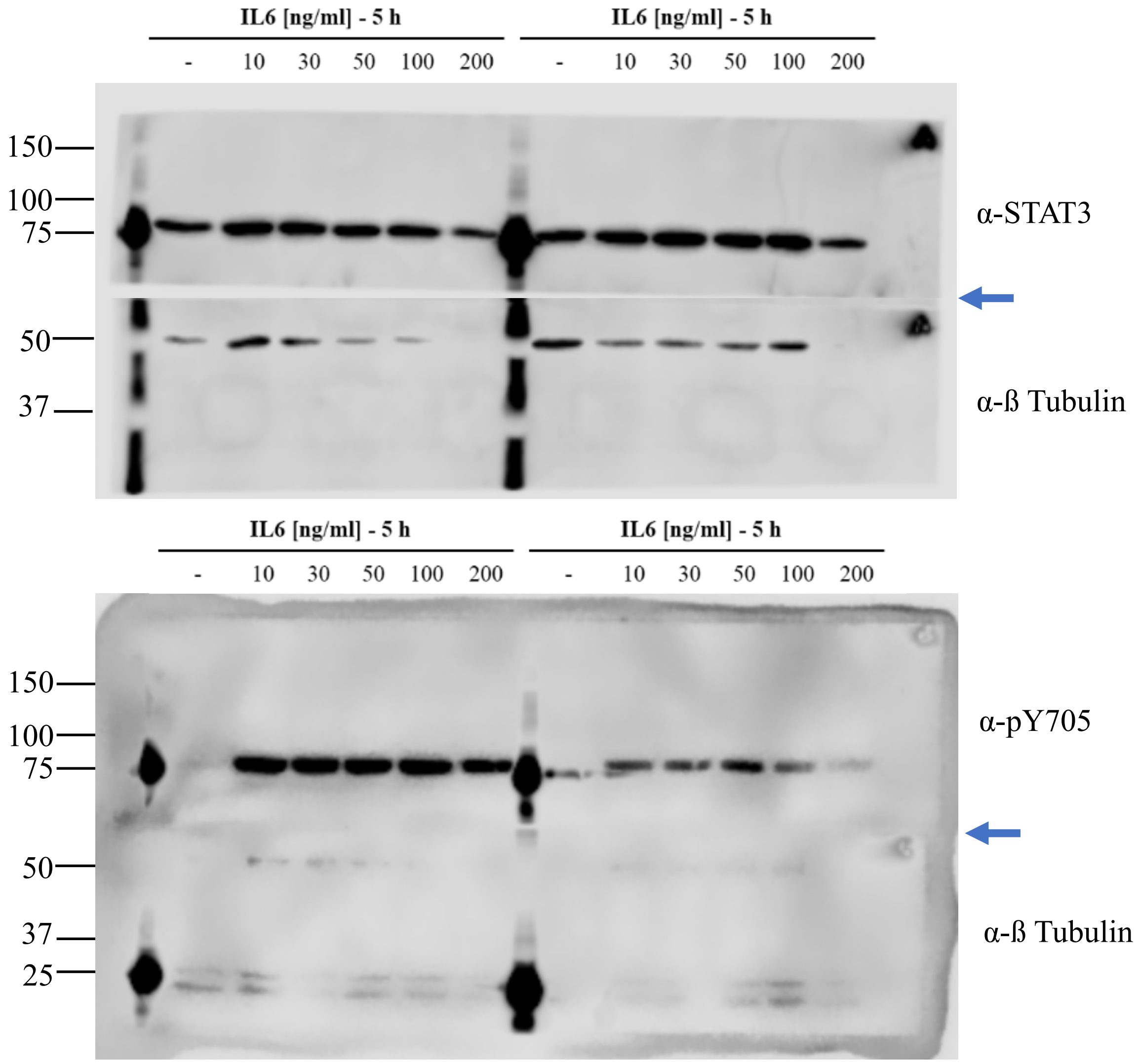

Replicate 1 Replicate 2

4. Supplementary Figure 1D

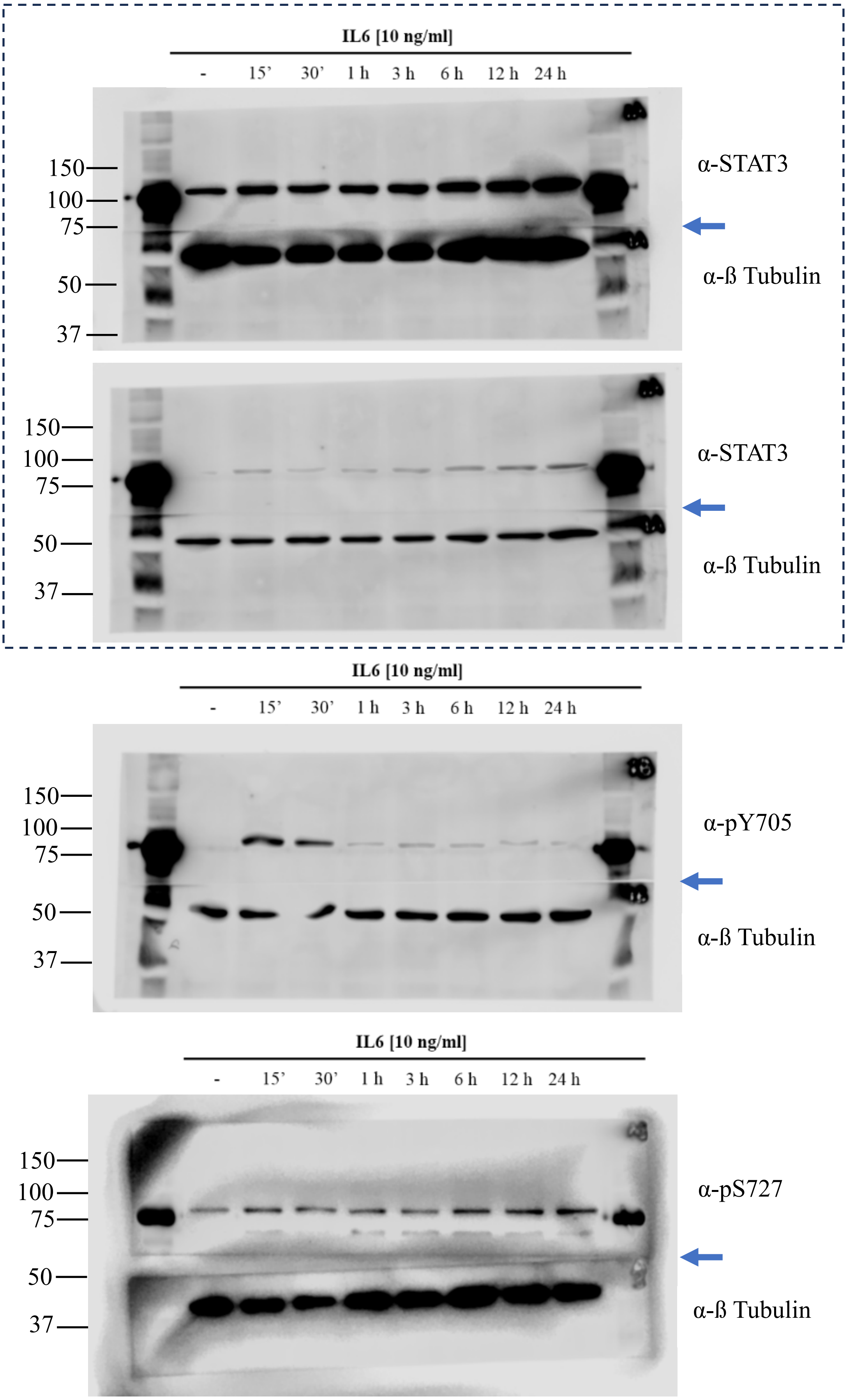

Replicate 1

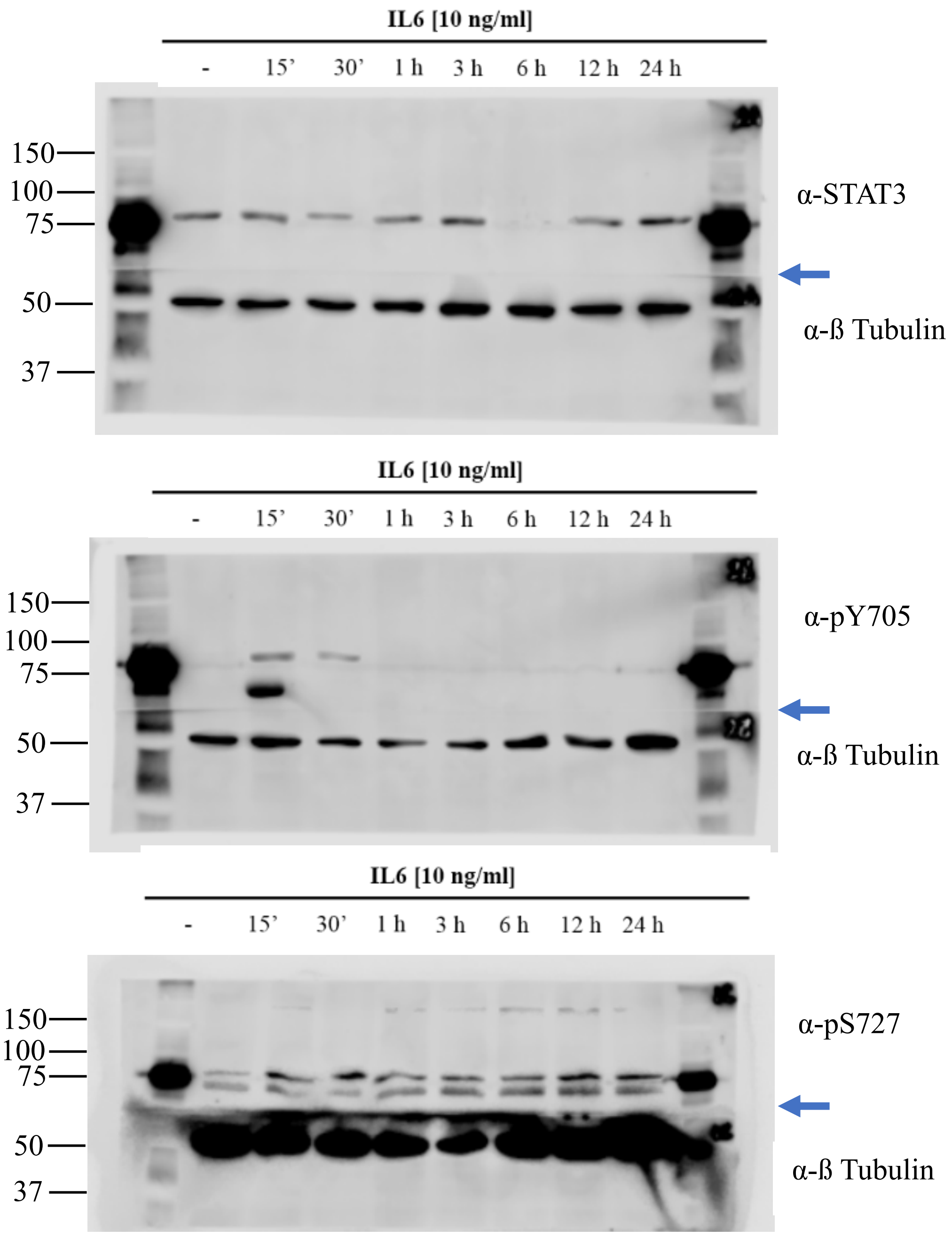
IL6 [10 ng/ml]
- 15' 30' 1 h 3 h 6 h 12 h 24 h
150
100
75
50
37
α-STAT3
α-ß Tubulin
IL6 [10 ng/ml]
- 15' 30' 1 h 3 h 6 h 12 h 24 h
150
100
75
50
37
α-pY705
α-ß Tubulin
IL6 [10 ng/ml]
- 15' 30' 1 h 3 h 6 h 12 h 24 h
150
100
75
50
37
α-pS727
α-ß Tubulin

Replicate 2

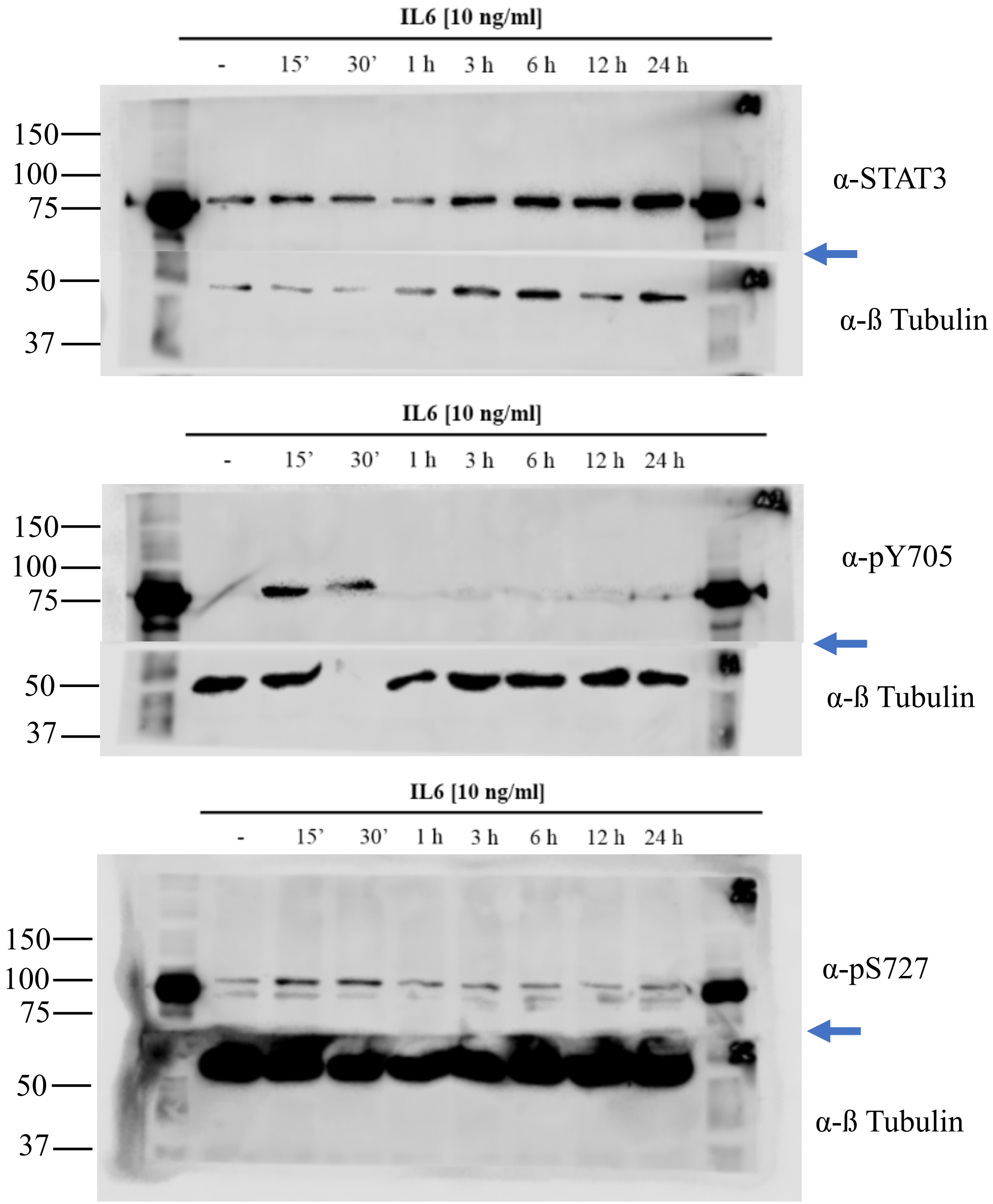
IL6 [10 ng/ml]
- 15’ 30’ 1 h 3 h 6 h 12 h 24 h
150
100
75
50
37
α-STAT3
α-ß Tubulin
IL6 [10 ng/ml]
- 15’ 30’ 1 h 3 h 6 h 12 h 24 h
150
100
75
50
37
α-pY705
α-ß Tubulin
IL6 [10 ng/ml]
- 15’ 30’ 1 h 3 h 6 h 12 h 24 h
150
100
75
50
37
α-pS727
α-ß Tubulin

Replicate 3

5. Supplementary Figure 1E

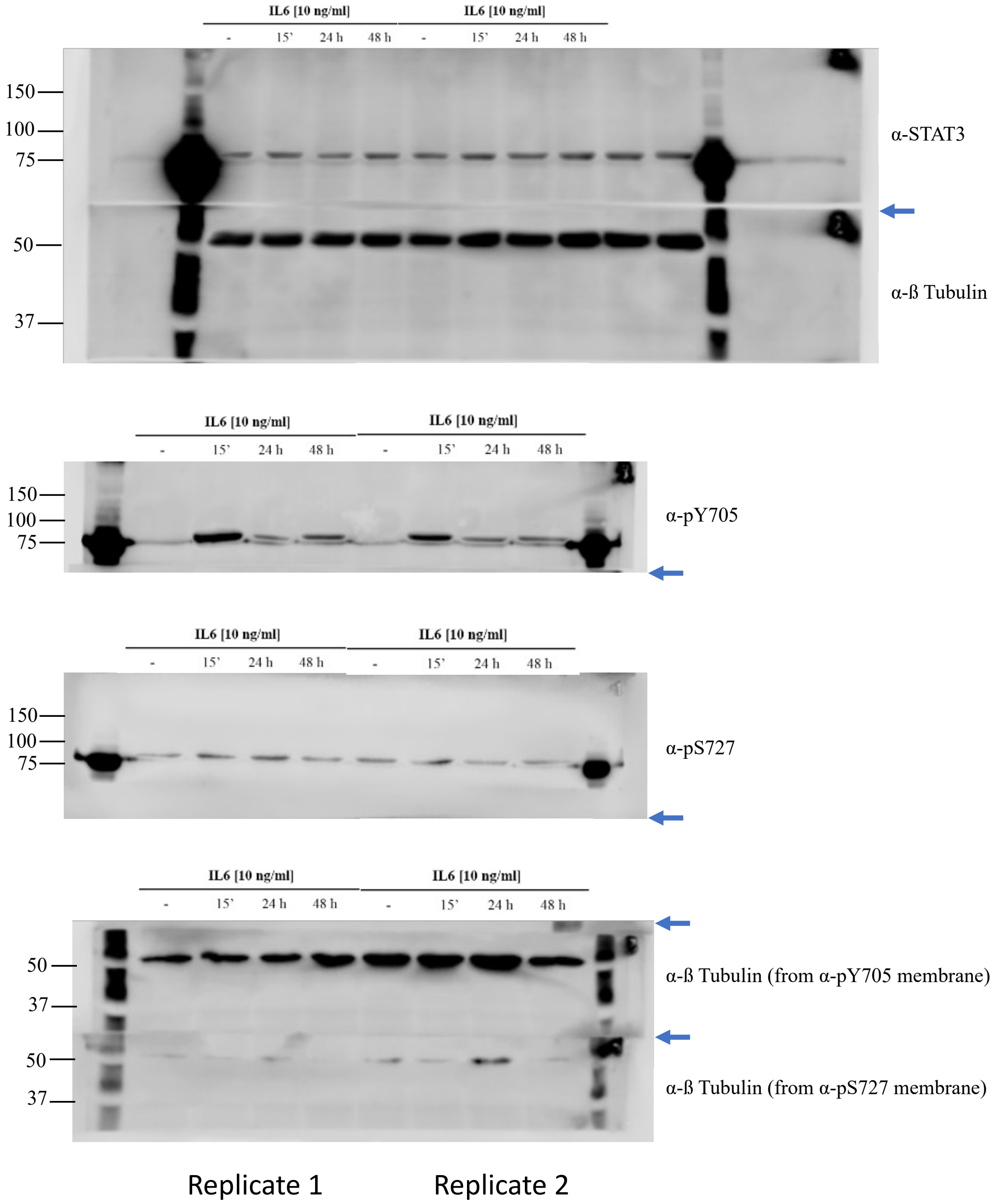

6. Supplementary Figure 2A

Full blots for each antibody are included.

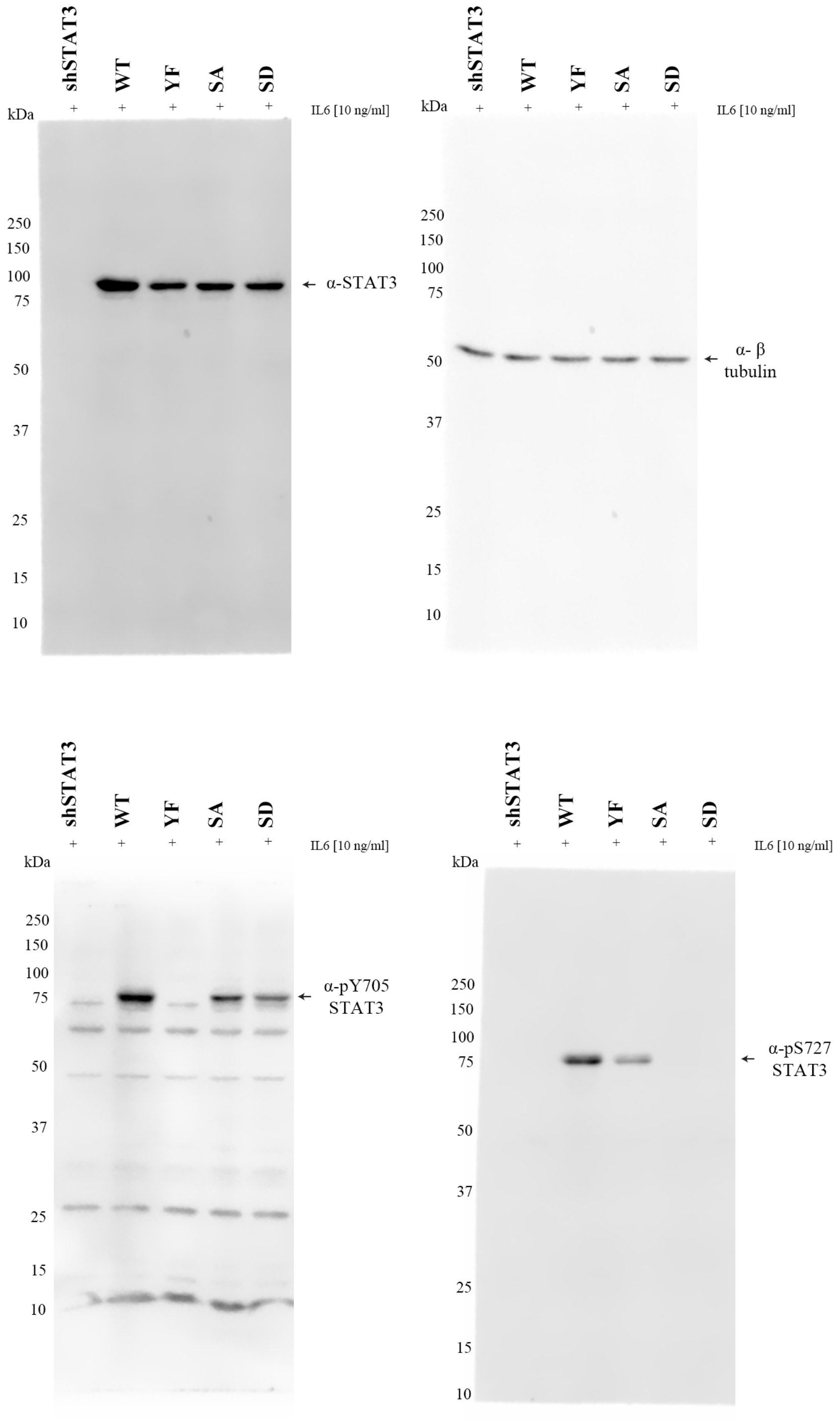

Supplement: Supplementary file 1 — Supplementary Information. [file 41598_2023_46628_MOESM1_ESM.pdf]
